# Evaluation of pulse crops’ functional diversity supporting food production

**DOI:** 10.1038/s41598-020-60166-4

**Published:** 2020-02-25

**Authors:** Julie Guiguitant, Denis Vile, Michel Edmond Ghanem, Jacques Wery, Hélène Marrou

**Affiliations:** 1 Montpellier SupAgro, INRAE, CIRAD, IAMM, Univ Montpellier, SYSTEM, F-34060 Montpellier, France; 2 INRAE, Montpellier SupAgro, Univ Montpellier, LEPSE, F-34060 Montpellier, France; 3 University Mohammed VI Polytechnic, AgroBioSciences, Benguerir, Morocco; 4 International Center for Agricultural Research in the Dry Areas (ICARDA), Cairo, Egypt; 5 Montpellier SupAgro, Univ Montpellier, F-34060 Montpellier, France

**Keywords:** Natural variation in plants, Plant breeding, Plant ecology, Plant physiology, Agroecology

## Abstract

Pulses, defined as legumes which produce dry seed used for human consumption, are plants of great agronomic value, at the food system level as much as the field level but their diversity has been largely underused. This study aimed at analyzing existing data on cultivated pulse species in the literature to provide a broad and structured description of pulses’ interspecific functional diversity. We used a functional trait-based approach to evaluate how pulse diversity could support food production in agroecosystems constrained by low water and nutrient availability and exposed to high weed pressure. We gathered data for 17 functional traits and six agroecosystem properties for 43 pulse species. Our analytical framework highlights the correlations and combinations of functional traits that best predict values of six agroecosystem properties defined as ecosystem services estimates. We show that pulse diversity has been structured both by breeding and by an environmental gradient. The covariance space corresponding to agroecosystem properties was structured by three properties: producers, competitors, stress-tolerant species. The distribution of crop species in this functional space reflected ecological adaptive strategies described in wild species, where the size-related axis of variation is separated from variation of leaf morpho-physiological traits. Six agroecosystem properties were predicted by different combinations of traits. However, we identified ubiquitous plant traits such as leaflet length, days to maturity, seed weight, and leaf nitrogen content, that discriminated agroecosystem properties and allowed us to gather individual species into three clusters, representative of the three strategies highlighted earlier. Implications for pulses provisioning of services in agroecosystems are discussed.

## Introduction

Over the past decade, grain legumes used for human consumption – pulses – have been receiving a resurgence of interest to meet agricultural challenges all over the world. Indeed, they are particularly important in human nutrition as sources of proteins, vitamins and minerals that complement a predominantly cereal-based diet^1,2^. At field level, their well-known ability to fix atmospheric nitrogen helps reducing energy consumption while making them particularly suitable for low-input systems. They are also a source of diversification in order to break disease, pest and weed cycles and optimize nutrient management in standard crop rotations^3^. Although site-specific environmental constraints may reduce cropping systems options, the diversity of pulse, when fully explored, may offer solutions to most cropping systems, from a biophysical point of view. Indeed, leguminous plants constitute the third largest family among flowering plants and contain no less than 18,000 species^4^. This diversity implies potentially highly variable responses to abiotic and biotic stresses^5^. However, the specific diversity of pulse is poorly represented in most cropping systems despite the growing demand from foodchains^6^. Most of pulse species could be defined as ‘neglected and underutilized species’ (NUS)^7,8^, i.e. useful plant species which are marginalized by researchers, breeders and policy makers^9^. While more than 80 pulse species contribute to the human diet, the FAO database includes only 11 of these^10^. They are, most of the time, locally important crops maintained by cultural preferences and traditional practices because they are nutritionally rich^11,12^ and adapted to harsh environments unfit for other crops where they can still provide sustainable productions^13^. However, local perceptions often differ from global priorities, mostly for economic reasons, as a result NUS such as most legumes species, are not included in large breeding efforts, dissemination programs, orforesight studies. This lack of attention creates a risk of continuous erosion of expertise and genetic resources that could lead to a further limitation of development options aimed to reduce the perceived riskiness of such crops^14^. Limitations to a broader cultivation and use of pulses could be attributed to their weak recognition in food system and markets’ bottom line^15^. However, as recent articles^16^ demonstrate, diversity is a key component of a sustainable diet (Healthy Diet for Healthy Planet^17^).

Beyond food security, the diversification of cropping systems with legumes could globally improve agroecosystem functioning^18^. Ecological research has demonstrated that ecosystem processes are determined by specific diversity. Increasing plant species richness may have a greater impact on ecosystem functioning at low diversity levels^19^. This suggests that, in cropping systems composed of a few species, small increases in biodiversity using legume crops can have a significant effect on ecosystem processes such as productivity or nutrient cycling^20^. Effects of biodiversity on agroecosystem properties can be evaluated through taxonomic diversity measures or, more recently, through plant functional traits^21–23^. The latter approach has the advantage of taking into account the significant differences between species, unlike “plant functional types” (PFTs), which often considers legumes as a homogeneous functional group^22,24^. In light of these considerations, it is essential to help maintain and use pulse genetic resources, to ensure sustainable development and use by present and future generations, in order to take full advantage of the services they offer.

This study aims at synthetizing and structuring current knowledge on the diversity of cultivated pulses at the interspecific level. Rather than offering practical solutions to farmers, the purpose of this study is to provide a base map of the functional trait space of pulses and its (possible) relationship with individual or combined ecosystem service provision. We choose to do this evaluation through the plant functional traits approach as defined earlier^21–23^. We assume that this approach could help to identify possible strategies by which multiple agroecosystem properties can be jointly controlled for a better ecosystem services provision, which is a crucial aspect of agroecosystem management. Here we define agroecosystem properties as traits or processes measured or calculated at the crop level^25^, such as biomass or the leaf area index. To this end, we gathered several functional traits^26^ in the literature to describe pulse species. Selected traits were characteristics of an individual plant or plant organ, which can be morphological, physiological or phenological and that are supposed to have direct or indirect effects on agroecosystem properties. Since ecosystem services are still poorly described, we focused on six agroecosystem properties hypothesized to be good indicators of three specific ecosystem services related to food provision in a low-input systems: (1) yield under dry conditions; (2) nitrogen fixation; and (3) competitiveness toward weeds. We discuss the potential of this approach to orientate breeding efforts and elicit conservation programs when further site specific and agroeconomic evaluations, that are essential to support actual integration of pulse diversity into farmer’s fields, would have been performed.

## Results

### Variability of pulse crops agroecosystem properties

**Figure 1:**
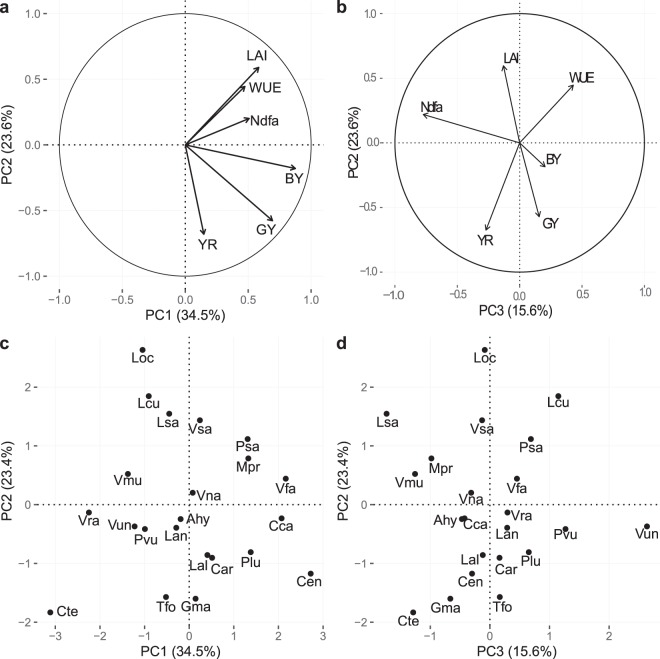
Principal component analysis (PCA) performed on six agroecosystem properties collected for 22 species of pulses. Visualization of the variables and correlation circles on (**a**) PC1-PC2 and (**b**) PC2-PC3 planes. (**c**,**d**) Individual species projection on the first three axes of the PCA. See Table 2 and Supplementary Table S1 for abbreviations of agroecosystems properties and Supplementary Table S1 for abbreviations of species.

The first three principal components (PC) altogether explained 74% of the variability of agroecosystem properties with the first PC (33%) strongly positively associated with biomass yield (BY) and grain yield (GY) and to a lesser extent %Ndfa (percentage of nitrogen derived from atmosphere) (Fig. 1a). This axis opposed high yield producers like *Canavalia ensiformis* (GY 2.53 ± 2.8 t ha^−1^, BY 9.79 ± 2.29 t ha^−1^) to poor yield providers like *Cymopsis tetragonoloba* (GY 1.01 ± 1.3 t ha^−1^, BY 1.42 ± 0.5 t ha^−1^), respectively representative of the phaseolids clade (warm season legume) and sister the indigoferoid clade. The second PC axis (24%) opposed LAI (leaf area index) to percentage of yield reduction due to weeds (%YR) and GY (Fig. 1a), but the correlations between the second PC axis and respectively, LAI and %YR were both low (*r* = −0.15, *P* = 0.5; Supplementary Table S2). %YR was also found to be only slightly correlated to GY (*r* = 0.27, *P* = 0.22). This could suggest that LAI alone is not enough to predict species ability to produce high yield under strong weed competition. The second axis opposed *Lathyrus ochrus* (LAI 4.1 ± 0.3, %YR 5%) and *Lens culinaris* (LAI 3.9 ± 2, %YR 2 ± 1%), both representative of Galegoid clade, to *Cyamopsis tetragonoloba* (LAI 1.4, %YR 79 ± 6%) and *Glycine max* (LAI 2.7 ± 1, %YR 60%), representative of the phaseolids clade. The third axis explained 16% of the variability and opposed WUE (water use efficiency) and %Ndfa (Fig. 1b). As expected, GY and BY were significantly correlated (*r* = 0.69, *P* < 0.001; Supplementary Table S2). PCA results associated %Ndfa to yield on the first axis, yet it was only slightly correlated to BY (Fig. 1; *r* = 0.22; Supplementary Table S2). This property (%Ndfa) along with WUE is well represented on axis 3 which opposed (Fig. 1c) *Vigna unguiculata* (%Ndfa 24.7 ± 0.5%, WUE 8.3 ± 10), representative of phaseolids clade, to *Lathyrus sativus* (%Ndfa 93.3 ± 3.5%, WUE 6.3 ± 3.1), representative of Galegoid clade. Pearson’s correlation coefficient showed a very slight positive, but not significant, correlation of WUE with LAI (*r* = 0.35, *P* = 0.11). Quality of the representation of WUE in the PCA was low; the property was slightly associated with the three axes with low explain of variation explain on each axis, and it was also slightly but not significantly correlated with BY (r = 0.27, *P* = 0.22).

The projection of individual species in the space of covariation showed that species characterized by high LAI and good tolerance to weed infestation (*Lathyrus ochrus*, *Lens culinaris*, *Lathyrus sativus*, *Vicia sativa*), are relatively unproductive species with GY varying from 0.6 to 1.1 t ha^−1^ and BY from 2.3 to 3.4 t ha^−1^. Moreover, they seemed relatively adapted to nitrogen deficiency and drought. Except for *Lens culinaris*, which showed a low nitrogen fixation efficiency, %Ndfa varied from 69% to 93%, while WUE varied from 6.05 to 7.5 kg ha^−1^ mm^−1^. However, relatively unproductive species could be sensitive to weed infestation, especially as they show a lower WUE (*Vigna radiata*, *Vigna mungo*) or a lower %Ndfa (*Vigna unguiculata*).

Productive species (GY from 1.1 to 2.5 t ha^−1^ and BY from 3.9 to 10.6 t ha^−1^) can be sensitive or insensitive to weed infestations (28 to 83% of yield reduction), and this ability cannot be related to nitrogen or water adaptations. However, productive species, except for *Phaseolus lunatus*, show a high values of WUE (from 5.2 to 10.4 kg ha^−1^ mm^−1^) and %Ndfa (from 61 to 82%).

*Cyamopsis tetragonoloba* is found apart from every other species in the space of covariation as it showed low values on every agroecosystem property.

### (Co-)variations of functional traits

**Figure 2:**
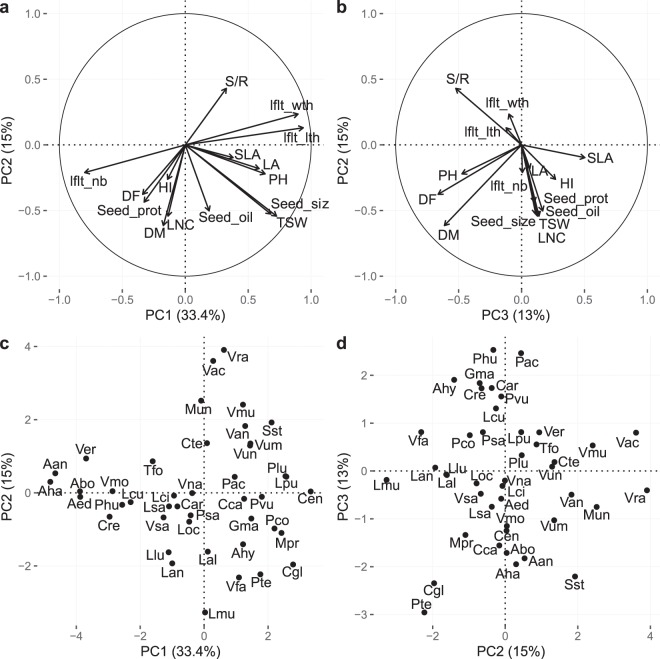
Probabilistic PCA performed on 15 functional traits collected for 43 pulses species. Visualization of the variables and correlation circles on (**a**) PC1-PC2 and (**b**) PC2-PC3 planes. Individual species projection on the first three axes of the PCA (**c**,**d**). See Table 2 and Supplementary Table S1 for abbreviations of agroecosystems properties and Supplementary Table S1 for abbreviations of species.

The first three PC axes of the PPCA (probabilistic principal component analysis) performed on functional traits collected for the 43 species explained 61% of total variance (Fig. 2). PC1 (33%) was associated with morphological traits, especially leaf traits (e.g. leaflet length, leaflet width, leaflet number, leaf area, plant height, and to a lesser extend seed diameter and seed weight). This axis opposed small plants such as *Astragalus sp*. (i.e. *A*. *hamosus*: seed diameter 2.1 ± 0.4 mm; TSW 17.7 ± 18.9 g; 21 ± 4.2 leaflets; leaflet length 9.8 ± 1.0 mm; plant height 55.9 ± 4.1 cm, LA 48.3 ± 19.6 cm^2^) to large plants such as *Canavalia sp*. (i.e. *C*. *gladiata*: seed diameter 23.3 ± 10.4 mm; TSW 1007.5 ± 382.3 g; tri-foliate; leaflet length 137.5 ± 72.2 mm; plant height 432.9 ± 186.8 cm; LA 143.7 ± 15.9 cm^2^). The number of leaflets is characteristic of the species’ origin; more than half (*n* = 26) of the species are trifoliate, most of them being tropical legumes except for a small number of Mediterranean species (*n* = 5) with a number of leaflets ranging from 1 to 4 leaflets per leaf. The remaining (*n* = 17) have many leaflets, ranging from 5 to 24; this was the case of Mediterranean and European species only. Number of leaflets showed significant negative correlation to leaflet length (*r* = −0.81, *P* < 0.001; Supplementary Table S3) which ranged from 9.8 ± 1.0 mm (*Astragalus hamosus*) to 137.5 ± 72.2 mm (*Canavalia gladiata*). Plant height (PH) ranged from 11.3 cm (*Cicer reticulatum)* to 432.9 ± 186.8 *(Canavalia gladiata)* and averaged 103.1 ± 91.8 cm. LA ranged from 21.11 cm^2^ (*Vicia ervilia*) to 6885.4 ± 5324.5 cm^2^ (*Psophocarpus tetragonolobus*) and was positively correlated to all leaflet traits and PH; leaflet number (*r* = −0.56, *P* < 0.001), leaflet length (*r* = 0.61, *P* < 0.001), leaflet width (*r* = 0.55, *P* < 0.01) and PH (*r* = 0.44, *P* < 0.01; Supplementary Table S3).

PC2 (15%) was strongly associated with seed traits such as thousand seed weight (TSW), seed diameter and seed oil and protein content (Fig. 2a). This axis opposed species with small, low quality seeds (i.e. *Vigna radiata* oil content 0.97 ± 0.2%; protein content 25.2 ± 1.8%; seed diameter 2.75 ± 1.8 mm; TSW 39.9 ± 11.1 g) to species producing larger seeds with higher oil and protein content (i.e. *Lupinus mutabilis* oil content 18.2 ± 5.2%; protein content 45.4%; seed diameter 11 ± 4.5 mm; TSW 286.75 ± 171.2 g) (Fig. 2c). Seed diameter ranged from 2.1 ± 0.3 mm (*A*. *annularis*) to 23.3 ± 10.4 mm (*C*. *gladiata*) with a mean of 7.8 ± 4.5 mm. TSW was closely correlated with seed diameter (*r* = 0.87, *P* < 0.001; Supplementary Table S3) and ranged from 12.8 ± 3.9 g (*Trigonella foenum-graecum*) to 1178 g (*Phaseolus coccineus*) and averaged 255.4 ± 294.1 g. Seed protein content ranged from 10.9% (*Canavalia ensiformis*) to 45.4% (*Lupinus mutabilis*) and was moderately correlated to seed oil content (*r* = 0.45, *P* < 0.01; Supplementary Table S3) which varied greatly from 0.4% (*Vigna angularis*) to 47.1 ± 1.9% (*Arachis hypogaea*) but with a dominance of low values (mean: 4.8 ± 8.4%).

The third PC axis (13.7%) opposed shoot/root ratio (S/R), days to maturity (DM), days to flowering (DF) and PH to specific leaf area (SLA) and leaf nitrogen content (LNC) (Fig. 2b). It opposed species with fast strategies for nutrient acquisition and use (high LNC and SLA) as *Phaseolus acutifolius* (DM 85 ± 26 days, DF 33 ± 9 days, LNC 43.5 mg g^−1^, SLA 420 cm² g^−1^) to species with slower strategies mostly based on their reproductive timing (DM and DF) such as *Astragalus boeticus* (DM 177 ± 3 days, DF 115 ± 5 days, LNC 19.3 ± 13.1 mg g^−1^, SLA 100 ± 25.3 cm² g^−1^) or *Canavalia gladiata* (DM 202 ± 9 days, DF 122 ± 9 days, LNC 23 mg g^−1^, SLA 253. cm² g^−1^). S/R ratio ranged from 1.2 ± 1.2 (*Phaseolus acutifolius*) to 19.4 ± 2.1 (*Sphenostylis stenocarpa*), its coordinate on the third axis was positive but S/R was not significantly correlated to DM and DF. Yet, it was negatively correlated to LNC (*r* = −0.4, *P* < 0.01) and positively to leaflet width (*r* = 0.52, P < 0.001). DM ranged from 79.5 ± 29.8 days (*Vigna radiata*) to 255 ± 148.5 days (*Lupinus mutabilis*) and was strongly correlated with DF (*r* = 0.69, *P* < 0.001) which ranged from 30 ± 35 days (*Cyamopsis tetragonoloba*) to 139.25 ± 30.6 days (*Astragalus hamosus*). SLA ranged from 39.2 ± 33.2 cm^2 ^g^−1^ (*Psophocarpus tetragonolobus*) to 537.2 ± 97.3 (*Lablab purpureus*). LNC ranged from 19.3 ± 13.1 (*Astragalus sp*.) to 60.2 ± 2.4 (*Lathyrus sativus*).

### Relationships between functional traits and agroecosystem properties

**Figure 3:**
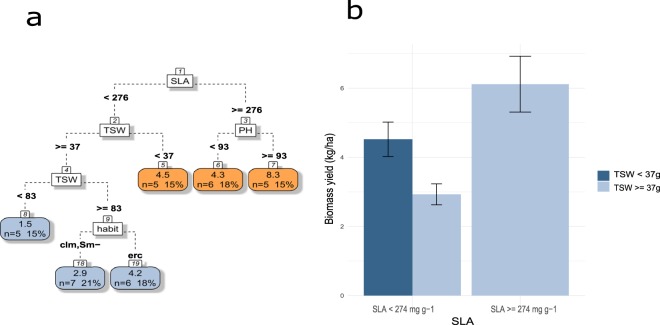
(**a**) CART regression tree for prediction of biomass yield of pulses species (n = 37). Root nodes represent single input variables (functional traits) and related split point used to make the prediction. Leaf nodes contain mean, number and percentage of observations of the predicted variable. (**b**) Mean and standard error of nodes 2 and 3. See Table 1 for abbreviations.

CART analysis showed that grain legume species with SLA above 274 cm^2^ g^−1^ (average 245.5 ± 105.4) produce more biomass (Fig. 3). BY is maximal (8 t ha^−1^) for high SLA combined with TSW above 37 g (which concerns most of the species as only nine species have smaller seeds), and PH above 83 cm (not far from the average 103.1 ± 91.8). BY around 4–5 t ha^−1^ can be achieved by small species (<83 cm) with high SLA, and doesn’t seem to be related to seed weight. Low SLA species can still achieve intermediate BY if they have small seeds. Among the lowest biomass producers (small SLA, big seeds) erect species and tall species have higher BY.

**Figure 4:**
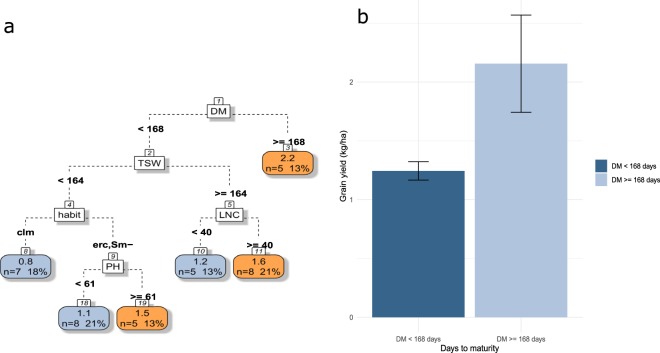
(**a**) CART regression for prediction of grain yield of pulses species (n = 39). Root nodes represent single input variables (functional traits) and related split point used to make the prediction. Leaf nodes contain mean, number and percentage of observations of the predicted variable. (**b**) Mean and standard error of nodes 2 and 3. See Table 1 for abbreviations.

GY is maximal (2.2 t ha^−1^) for species which take more than 168 days to mature (average 135.0 ± 38.4) (Fig. 4). In early maturing species, GY is maximized when LNC is higher than 40 mg g^−1^ (average 38.4 ± 12.1) in species that produce big seeds (TSW > 164 g). In small-seeded species (TSW < 164 g), there is no relation between LNC and GY whereas erect habit generally leads to higher GY than climbing habit. Rapid crop cycle combined with small seeds generally leads to low GY with one exception for tall species (PH >= 61 cm) with erect habit.

**Figure 5:**
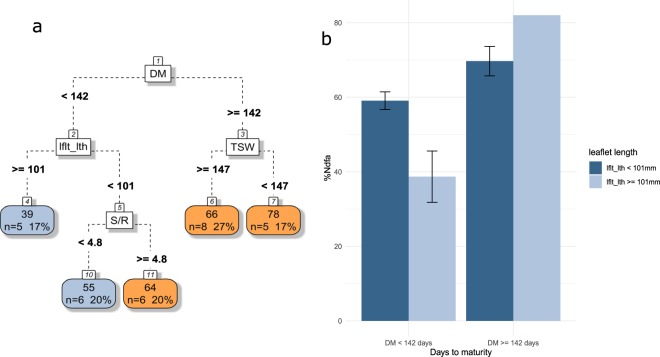
(**a**) CART regression for prediction of %Ndfa of pulses species (n=30). Root nodes represent single input variables (functional traits) and related split point used to make the prediction. Leaf nodes contain mean, number and percentage of observations of the predicted variable. (**b**) Mean and standard error of nodes 3, 4 and 5. See Tables 1 and 2 for abbreviations.

The number of days to maturity (DM) was also discriminant for %Ndfa. This agroecosystem property is indeed maximized (66–78%) for late maturing (DM >= 142 days) species, among which small-seeded species (TSW < 147 g) fix more nitrogen from the atmosphere than large-seeded species (TWS >= 147 g; Fig. 5). A slightly smaller amount of nitrogen fixation could also be reached by early maturing species with smaller leaflets length (<101 mm) and S/R specifically above 4.8. Yet, the advantage of a large S/R ratio was only observed in this situation, while early maturing species with long leaflets exhibit low %Ndfa.

**Figure 6:**
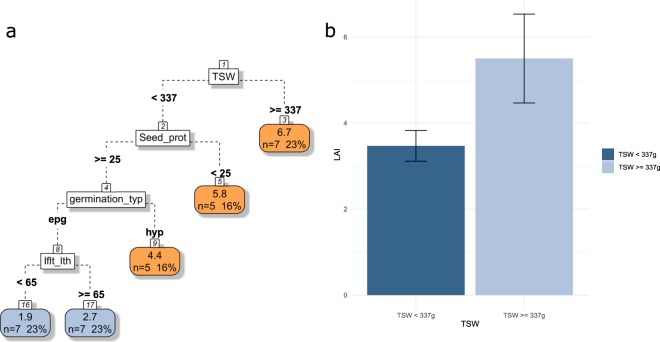
(**a**) CART regression for prediction of LAI of pulses species (n=31). Root nodes represent single input variables (functional traits) and related split point used to make the prediction. Leaf nodes contain mean, number and percentage of observations of the predicted variable. (**b**) Mean and standard error of nodes 2 and 3. See Tables 1 and 2 for abbreviations.

In our data analyses, LAI of pulses is sequentially smaller in small-seeded species that have high protein content in their seeds, an epigeal germination and small leaflets (Fig. 6).

**Figure 7:**
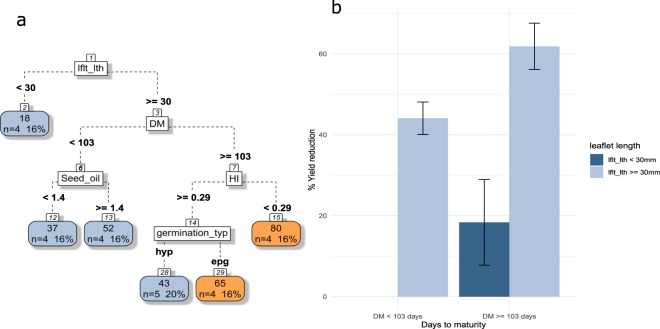
(**a**) CART regression for prediction of yield reduction of pulses species (n=25). Root nodes represent single input variables (functional traits) and related split point used to make the prediction. Leaf nodes contain mean, number and percentage of observations of the predicted variable. (**b**) Mean and standard error of nodes 2, 6 and 7. See Tables 1 and 2 for abbreviations.

Yield of species with very small leaflets (length < 30 mm) is less negatively impacted by weeds (Fig. 7). However, leaflet length under 30 mm is only encountered in four species over the 25 and two of them are under 10% YR which is extremely low compared to other species. For the remaining 21 species, DM was again a crucial trait. Short cycle (<103 days to maturity) leads to reduced yield losses due to weeds (under 60%). Yet, a long crop cycle duration combined with hypogeal germination and a better harvest index (HI) than average (>=0.29) also resulted in a similar percentage of yield loss due to weeds (43%). Among early maturing species, yield of those that have the highest oil content in seeds is less affected by the presence of weeds.

**Figure 8:**
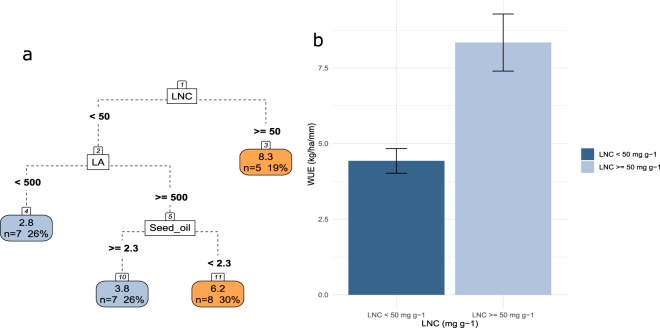
(**a**) CART regression for prediction of WUE of pulses species (n=28). Root nodes represent single input variables (functional traits) and related split point used to make the prediction. Leaf nodes contain mean, number and percentage of observations of the predicted variable. (**b**) Mean and standard error of the node 2 and 3. See Tables 1 and 2 for abbreviations.

WUE (8.3 kg ha^−1^ mm^−1^) is maximized in species with high LNC (>=50 mg g^−1^) (Fig. 8). While in species with low LNC those with small leaves have the lowest WUE, it is noticeable that in species with larger leaves could have a low WUE is significantly higher for those that have the lowest seed oil content.

We conducted a sensitivity analysis to control the effect of sampling on CART result. We considered 50 random samples containing 75% of the trait values collected in the database and repeated the CART analysis for each agroecosystem property. Sensitivity analysis revealed that trait value sampling would not affect the CART results significantly for all functions except WUE. Indeed, in more than 50% of the regressions, the top three segregating traits were found to be the same as when considering the average of collected values for each trait per species (data not shown).

A hierarchical clustering analysis classified pulses in three groups (Supplementary Fig. S1) with a range of agroecosystem property values that were in accordance with PCA results, that is, high BY and GY for cluster 1, high %Ndfa and WUE for cluster 2 and low LAI and high %YR for cluster 3. Details about the classification of species based on the values of the five top traits for property prediction (SLA, LNC, TSW, DM, leaflet length), and species cluster values for each agroecosystem property are given in Supplementary Materials (Supplementary Fig. S1).

## Discussion

In this study, we aimed at quantifying the pulse’ interspecific functional diversity and its relationships with the provisioning of ecosystem services, therefore providing a broad base map for future design and evaluation of diversified cropping systems. Quantification of ecosystem services is most often achieved through monetary evaluation, which requires many hypotheses that are mostly context dependent^27^. In addition, ecosystem services in agriculture are essentially those supporting or regulating production services, such as the three services we have targeted to support food production, by improving soil fertility, climatic limitation or pest regulation^28^. Quantifying ecosystem services as if they were independent from each other can be in vain. Instead, we focused on agroecosystem properties which were available in the literature and hypothesized as indicators of ecosystem services. We followed a functional traits approach to draw comprehension of how crop traits could influence these properties and, as a result, the overall service production of an agroecosystem. This approach explicitly assumes that relationships exist between traits as well as between agroecosystem properties in order to better decipher the trade-offs between services and their causes of variation. We have selected agroecosystem properties that were related to crop yield, water use efficiency, nitrogen fixation and ability to maintain yield under competition with weeds.

### Plant production strategies among pulse species

Collected values for the six agroecosystem properties revealed three main axes of variation. The first axis sorted pulses based on their biomass and grain production level. The second axis was mostly related to response to competition, especially against weeds, while the third axis sorted pulses based on their ability to fix nitrogen. Despite its wide variation among pulses, water use efficiency was poorly represented and occupied an intermediate position in this space of variation. These results might highlight segregation between species that are adapted to productive versus unproductive or competitive environments. This is consistent with Grime’s CSR triangle theory that predicts plant strategies are selected along environmental gradients of resource availability, stresses and perturbation^29,30^. Grime’s theory highlighted that these gradients might have selected optimized trade-offs between phenology or development and resource acquisition in plants. This results in a CSR classification based on a combination of three extreme plant strategies: competitors (C), stress-tolerant (S), and ruderals (R)^31^. When applied to Mediterranean pasture legumes such as *Medicago sp*.^32^, *Trifolium* sp.^33^ or *Lupinus* sp.^34^, this theory was in accordance with the variation of traits related to water acquisition and use, and highlighted contrasted plant adaptations to drought^35^. In the species set surveyed here, the third axis might partially represent species adaptation to stressful environments with low levels of nitrogen and water. The first axis of the PCA sorted the species on their productivity ability selected in potentially highly productive environments such as fertilized and irrigated crop systems associated with limited competition due to recurrent perturbations (mechanical or chemical destructions). This axis could be associated to R-strategies. However, productive species could be more or less sensitive to competition mostly depending on their ability to intercept light as suggested by the second axis of the PCA. This competitive ability might have been selected in highly productive and crowded environments and be related to C-strategy. However, agroecosystem are ruderal environments: pulses as crop species have a peculiar evolutionary history compared to uncultivated species due to their breeding background. They played an important role in human nutrition and thus might have been more often managed under non-limiting conditions (irrigation and fertilization) and would exhibit high grain production levels compared to species with less importance and that were predominantly used for feed. Thus, it is more likely that axes 1 and 2 show classification of competitive ability inside ruderal strategies.

Finally, species that were considered in this study came from a wide range of geographic origins and have therefore been subject to a great diversity of climates, farming practices and resources along their history, leading to contrasted adaptations to stressful conditions, where nitrogen fixation may be determining in competitive capacity. In the current context of climate change and reduction of resource availability, species that are able to maintain relatively high productivity under stressful conditions could be valuable for the provision of food.

### Patterns of trait covariations and plant production strategies

Our results showed trait covariation patterns consistent with those predicted and observed by common Plant Ecological Strategies Schemes (PESS) previously described in wild species^29,36–40^. Leaf, seed or growth strategy are major components of CSR strategy variation. Unfortunately, we were unable to position pulse crops in the Grime’s CSR triangle (e.g. using the computation^31^ due to the lack of trait values. We found that seed and leaf traits exhibited the greatest variability among the 43 pulse species, in accordance with Westoby’s LHS (leaf-height-seed) scheme^36^. The first PC axis was best explained by leaf size, leaf area and, especially, plant height, which is recognized as a good indicator of plant capacity for competitive dominance^36,41,42^. Variation of seed size and TGW was mostly associated to the second axis of our PCA but had an intermediate position in the first PC plan and indicated that the seed dimension axis defined by Westoby (1998), as an expression of the potential of dispersal and successful establishment of a species^43–45^, was marginally correlated to plant and leaf size dimensions in the studied pulses; this result was consistent with previous findings by Leishman^46^. The third dimension of the LHS scheme relates to the capacity of plants to exploit resource-rich and resource-poor environments. This dimension reflects the trade-off between “fast” traits that promote rapid resource acquisition and “slow” traits that promote resource conservation^47–49^. In the search for a single trait that captures the core of this axis, specific leaf area (SLA) is a leading contender^50–52^. However, our study shows that this trait contributed only marginally to the total trait variation among pulses through its contribution to the third PC axis. Part of pulse variability represented on the third PC axis was best represented by days to maturity and flowering, which can also be negatively related to plant capacity to rapidly acquire resources. As previously observed by Tribouillois^53^ for herbaceous Fabaceae crops, the relationship between SLA and LNC was similar to that observed across a large diversity of wild species^48,49^ although LNC values of N-fixer species were on average higher than non N-fixer species (see Supplementary Fig. S1). The total percentage of variability explained by the first three PCs (61%) was relatively low compared to what is usually observed in wild and cultivated species^48,49^. This could be attributed to the collection method of data and/or the peculiarities of these cultivated Fabaceae species.

### From trait profiles to services

Functional traits are directly or indirectly linked to ecological processes leading to agroecosystem properties. Several trait can be involved in one process and one trait can participate in several processes. Thus, a given trait can only predict ecosystem properties value as long as every other traits have fixed values and processes occur at a fixed rate. CART regressions were particularly relevant to overcome this issue. For example, our results show that high WUE efficiency could be achieved by plants with LNC over 50 mg g^−1^ or, alternatively, with low LNC if total leaf area was above 500 cm^2^. Similarly, late maturing species matched high yield, but early maturing species were still able to achieve GY up to 1.6 t ha^−1^ if they had high TSW (>=164 g) and LNC above 40 mg g^−1^. In addition, plant traits which are determinant for good performance in one property could be disadvantageous for another when they are combined with other traits. For example, long crop duration is in favor of GY and %Ndfa but could end up to 80% YR under weed infestation among species with large leaflets. It is therefore difficult to define one ideal combination of plant functional traits which would maximize all ecosystem properties. TSW participated in the prediction of most agroecosystem properties. However, we found that prediction of ecosystem properties did not rely on the central traits used for the quantification of each axis alone. Leaflet length was found more relevant than plant height to predict competitive ability against weeds and days to maturity a better predictor than SLA for prediction of pulse productivity as indicated by CART results for prediction of biomass and grain yield performances.

The first targeted ecosystem service was food production under dry conditions. The objective was not to differentiate pulse species by their drought resistancebut rather by their ability to maintain high production rate under drought, which is a more relevant trait for food production. WUE is a good candidate for that purpose even though it is not the only driver of effective use of available water or drought resistance. Over the 28 species considered here, high WUE was observed mainly for species with high LNC. LNC at anthesis and grain yield under drought have been previously found to be strongly linked^54^. Indeed, when nutrient uptake is limited by water availability^55^, remobilization of N from vegetative tissues becomes particularly important for grain growth^56^. Moreover, high LNC is usually associated to the “stay green” type. More especially in case of terminal drought, it has been shown that if LNC declines to a critical threshold, leaf senescence will set up^54,57^. It is therefore not surprising that N leaf status is very closely related to the longevity of photosynthetic organs^58,59^. In case of terminal drought, high LNC maintains photosynthetic capacity for longer, sometimes leading to higher grain yield, and allows greater N remobilization. Furthermore, species with low LNC were more likely to have high WUE if associated with large leaf area. Although large leaf area may cost more water loss, it is also possible that it would result in more remaining photosynthesis area at the end of the drought stress period, thus allowing to achieve higher yields. WUE was poorly represented in the PCA. Because of this positioning, it could hardly be associated with other properties. Understanding its interaction with grain yield could have been particularly interesting for breeding purposes. Grain yield was not predicted with the same set of traits than WUE except for LNC (LNC is important for pod filling whatever water conditions are). Seed weight (TSW) was a good predictor of grain yield. Indeed, seed size is expected to be positively correlated with seedling biomass^60,61^, plant height and reproductive effort^62^. Seed size might also have been a result of agronomic selection such as erect habit, characteristic of most high yielding species and a trait highly related to domestication^63^.

Cropping systems that incorporate grain legumes have been shown to strongly decrease N fertilizer rates (by 13–30% for wheat and 49–61% at the rotation level) through nitrogen fixation^64^. However, legume species are not equivalent in their ability to supply exogenous nitrogen to the system. In our study, the percentage of nitrogen fixed varied substantially across species (from 23.75 ± 15.34% to 93.33 ± 3.51%). In addition to species inherent capacity to fix N, this variation could be attributed to nutritional factors, environmental conditions, rhizobia strains or host characteristics^65–67^. Since the data collected focused on plant traits, an incomplete picture of what drives nitrogen supply might have been obtained. CART regression showed that high nitrogen supply was mostly achieved by late maturing species. This result is in accordance with previous studies^68,69^. Nitrogen fixation is more favorable for grain yield during the latter part of the growth cycle of a legume than it is during early growth^68,70^. These factors account for the superior symbiotic performance of late maturing bean cultivars. In addition, any process that increases growth rate also increases tissue turn-over and loss of carbon, nutrients and water, alongside with decreasing allocation to storage^29^ and, thus, possible allocation to nodules. Our results highlighted small leaflets as a secondary trait involved in high nitrogen supply. The presence of compound leaves is a widespread trait of legume species and leaves divided into small leaflets appears to be a frequent component of ecological strategies emphasizing a productive photosynthetic apparatus^71,72^. In theory, nitrogen fixation may require/mobilize 10 to 20% of the total plant photosynthesis^70,73^. Thus, high photosynthetic productivity might allow a greater allocation of photosynthetic compounds to nodules. N_2_ fixation has been shown to be closely synchronized with the rate of supply of translocate from the shoot to nodules^74,75^. Here we considered the proportion of nitrogen fixed by a plant (%Ndfa), independently of biomass production aspect, as an indicator of nitrogen supply. This trait is related to plant N fixation efficiency andits ability to grow in nitrogen-poor environments. However, biomass yield is hardly dissociable from nitrogen fixation as it is a process driven by N demand^76,77^. CART regression showed that high biomass species had large SLA. High SLA might allow (given favorable growth conditions) a shorter payback time on a gram of dry matter invested in a leaf^78^, therefore improving production rate.

The last targeted ecosystem service was competitiveness toward weeds, which is a service that is particularly relevant when considering legumes as cover crops^79^ or growing grain legumes without herbicide use. In addition to providing an indicator for weed control, competitiveness towards weeds might also be a good indicator for the management of plant species interactions in intercropping^80^. Higher level of competition, however, might also induce competition for nutrient or water supply^81^. Our results show that species with a higher WUE were more insensitive to weed competition. It has also been shown in previous studies that root competition for soil resources and shoot competition for light are occurring simultaneously and are interrelated in pea-barley intercropping^82^. Yet, in our study, %YR was mostly found correlated with LAI indicating that light competition might be more important. Many ecological studies have pointed out that competition is mostly encountered in highly productive environments. Indeed, when soil resources are more abundant, light might become the limiting resource^41^. LAI is higher for large seeds with hypogeal germination. As for biomass, grain yield and nitrogen fixation, LAI can be related to seedling vigor. In addition, our results show that yield was less likely to be impacted by weeds for early maturing species with small leaves; which are also species with a high nitrogen fixation ability. Indeed, in our study, small leaflets allowed early maturing species to achieve almost similar %Ndfa level than late maturing species. This suggests that the use of alternative nitrogen source is also an important component of pulses competitive ability. Some studies highlighted that nitrogen fixation increased in case of interaction with non-fixing species^80,83^ suggesting competition for nitrogen might disfavor pulse species which do not have the capacity to improve their fixation rate. In addition, small leaves are less expensive to produce^78^ and might be more efficient for light interception as it reduces the risk of auto-shading^63^. Beside leaflet size, early growth provides an important advantage to the crop for light acquisition as well as for nitrogen accumulation through a faster and deeper root growth^84^.

### Limits

The approach followed in this study has some limitations, which could potentially limit the extent of our conclusions. Due to the relatively low and uneven number of values reported across traits and species, we had to consider aggregated values for each species and each trait. After comparing different summary statistics, mean was found to be the best aggregating function (compared to median, maximum, and minimum). Standard deviation was judged non reliable to estimate the intraspecific variation, due to the relatively low and uneven number of values for evaluating intra-specific variation. However, for each documented species, trait and agroecosystem properties have been measured on various genotypes or cultivars, which probably accounts for within species variability^85^. In addition, genera boundary are still discussed in grain legumes^83^. In this study, we relied on current taxonomy to delimitate species, which may have misled us into aggregating together trait values that actually belong to distinct species. This inaccuracy in identifying species may have contributed to some extent to blur limits between identified plant strategies. Data availability also led to some degree of incomplete database where some traits or species were more documented than others. We tried to overcome this weakness by imputing data through probabilistic principal component analysis, although we acknowledge that this estimation might have induced bias for the agroecosystem property’s prediction. Although non-optimal data availability and accuracy may explain partly the large number of trait combinations predicting each agroecosystem property, our analysis was solid enough to identify ubiquitous contributing traits and plant strategies for each targeted ecosystem service.

## Conclusion

Defining crop ideotypes through trait profiles for ecosystem service provision is tempting. However, our study suggests that the numerous correlations between traits and between agroecosystem properties themselves, and more importantly the inconsistency of trait combinations that best predict the different properties, may hinder it. Our analytical framework suggests that variation in agronomic performance and related ecosystem services, at an interspecific level among pulses, follow ecological strategies theories. However, further work will be required to explicitly take into account the effects of environmental changes as well as the role of intraspecific variation in such a diverse group. Although agroecosystem properties were predicted by multiple alternative combinations of traits it was possible to identify leaflet length, days to maturity, seed weight, and LNC as ubiquitous plant traits that discriminate ecosystem services provisioned by pulses. This discrimination was more or less consistent with adaptive strategies schemes since advantageousness of a trait was dependent on the ecosystem service under consideration. The achievement of a desired set of ecosystem services may require combining antonymic or negatively correlated properties which renders the identification of suitable traits pattern more complex, as more combinations become susceptible to be suitable.

## Material and Methods

### Approach

Our extensive literature review allowed to collect values for these six agroecosystem properties as well as a total of 17 traits for 43 pulses species. We discuss the combinations of pulses’ functional traits that may favor individual or combined ecosystem services provision.

### Data sources

We compiled a database from 327 published studies where at least one plant trait or agroecosystem property was measured on a pulse species (see complete reference list in Supplementary Table S4). We selected 17 functional traits measured at the sub-individual or individual level, based on data completeness (Table 1). We were able to document 43 pulse species on most of the traits selected (~5% of missing values) (Table 1). The complete list of covered species is provided in supplementary materials (Supplementary Table S1). Species covered the two clads that gather the majority of legume crops, namely Galegoid and Phaseoloid, which are often referred to as cool season and tropical season legumes, respectively^5^.We also gathered data on six agroecosystem properties measured at the supra-individual (population) level, but these data were less complete than the traits (~17% missing values) (Table 2). These six properties have identified relations with targeted ecosystem services. We considered that food provision under drought is a function of grain yield and water use efficiency (WUE) (Eq. 1). Nitrogen supply service is predicted to be tightly related to biological nitrogen fixation (BNF) which is a function of the percentage of nitrogen derived from atmosphere (%Ndfa) and biomass yield (BY) (Eq. 2). Finally, competition against weeds was assessed by grain yield loss when the crop is subject to competition which we supposed to be function of maximum crop leaf area index (LAI) divided by the time needed to reach the maximum LAI (Eq. 3).

1 $$Food\,service{s}_{drought}\leftarrow \,\frac{{\boldsymbol{Yield}}}{Tr}\times water={\boldsymbol{WUE}}\times water$$

2 $$Nitrogen\,supply\leftarrow BNF={\boldsymbol{biomass}}\times  \% {\boldsymbol{Ndfa}}\times \,[N]total$$

3 $$Weed\,interference\leftarrow {\boldsymbol{Yield}}\leftarrow f(\frac{{\boldsymbol{LAImax}}}{Flowering-Germination})$$

**Table 1:** Functional traits collected for 43 species of pulses.

Trait name	Abbreviations	Unit	% of missing value
Days to maturity	DM	Days	0%
Days to flowering	DF	Days	0%
Seed diameter	Seed_size	mm	0%
Number of leaflet	lflt_nb	number	0%
Leaflet width	Lflt_wth	mm	2.3%
Leaflet length	Lflt_lth	mm	2.3%
Plant height	PH	cm	0%
Habit		Erect, climbing	0%
Germination type		Epigeal, hypogeal	0%
Leaf nitrogen content	LNC	mg g^−1^	9.3%
Thousand seed weight	TSW	g	0%
Grain protein content	Seed_prot	%	0%
Grain oil content	Seed_oil	%	0%
Leaf area per plant	LA	cm²	18.6%
Specific leaf area	SLA	cm² g^−1^	16.3%
Shoot to root ratio	S/R	—	9.3%
Harvest index	HI	—	23.2%

**Table 2:** Agroecosystem properties collected for 43 species of pulses.

Agroecosystem function (services indicator)	Abbreviation	unit	% of missing data
Grain yield	GY	t.ha^−1^	2.3%
Biomass yield	BY	t.ha^−1^	7%
Water use efficiency	WUE	kg.ha^−1^.mm	25.6%
Percentage of nitrogen derived from atmosphere	%Ndfa	%	18.6%
Yield loss (due to weeds)	%YR	%	30.2%
LAI	LAI	—	16.3%

Data originated from very diverse sources (scientific literature, flora, crop guides, databases) since no study have reported data for all the traits for one species. For a given species, values for a given trait or a given agroecosystem property may differ depending on the genotype (G), environment (E) or cropping practice (M) that was applied to crop. Although GxExM effects add more variability to the dataset, it was impossible to separate it from the interspecific effect as no published literature would compare species as diverse as tropical perennial pulse species versus cool season short cycle pulse crops. To limit over-weighting of extreme values and sampling effect, we collected more than one value per trait and species and average was used as an aggregation function. Moreover, it has been demonstrated that species trait values are consistent enough to allow values to be used from different data sets (experiments, databases) to characterize local populations of species^86^.

### Data analysis

We initially focused on the linkages between agroecosystem properties, in order to get an overview of the potential trade-offs between services. A principal component analysis (PCA) was conducted on a reduced number of species (*n* = 22) still representative of the two clades that gather the majority of legume crops.

In order to describe the functional diversity of pulse, we performed a probabilistic principal component analysis (PPCA) on functional traits. This is a dimensionality reduction technique that analyzes data via a lower dimensional latent space^87^. It is often used when there are relatively low levels of missing values in the data or for multidimensional scaling.

To identify the best explanatory pulse functional traits for predicting agroecosystem functioning, we performed a classification and regression tree (CART) analysis. These prediction models are obtained using machine-learning algorithms that recursively partition the data space in order to fit the simplest prediction model within each partition. The resulting partitioning can be represented graphically as a decision tree^88^. Six trees were built to predict each agroecosystem property with trait values extracted from the PPCA. To control the effect of having only few trait values for some species and of GxExM interaction, we conducted a sensitivity analysis on the effect of trait value sampling on the results of the CART analysis. A hierarchical clustering was performed on the 43 species based on the traits revealed by CART regression as the best predictors of agroecosystem properties.

All statistical analyses were performed in the computing environment R 3.5.2^89^ using *pcaMethods* package^90^ and *rpart* package^91^.

## Supplementary information

Supplementary Information.
