# Supplementary material for: Evaluation of pulse crops’ functional diversity supporting food production
Source: Sci Rep. 2020 Feb 25;10:3416. doi: 10.1038/s41598-020-60166-4 (PMC7042262; doi:10.1038/s41598-020-60166-4)
Supplement: Supplementary file 1 — Supplementary Information. [file 41598_2020_60166_MOESM1_ESM.docx]

*Supplementary figures / tables*

**Evaluation of pulse crops’ functional diversity supporting food production**

Julie Guiguitant^1,2^, Denis Vile^2^, Michel Edmond Ghanem^3^, Jacques Wery^4^, Hélène Marrou^3,5^

^1^SYSTEM, Montpellier SupAgro, INRA, CIRAD, IAMM, Univ Montpellier, Montpellier, France

^2^LEPSE, INRA, Montpellier SupAgro, Univ Montpellier, Montpellier, France

^3^University Mohammed VI Polytechnic, AgroBioSciences, Benguerir, Morocco

^4^International Center for Agricultural Research in the Dry Areas (ICARDA), Cairo, Egypt

^5^Montpellier SupAgro, Univ Montpellier, Montpellier, France

**Corresponding authors**: Hélène Marrou (helene.marrou@um6p.ma), Denis Vile (denis.vile@inra.fr)

upplementary Table S1 : 43 species documented in this study

| Species | abbreviation |
| --- | --- |
| Arachis hypogaea | Ahy |
| Astragalus annularis | Aan |
| Astragalus boeticus | Abo |
| Astragalus edulis | Aed |
| Astragalus hamosus | Aha |
| Cajanus cajan | Cca |
| Canavalia ensiformis | Cen |
| Canavalia gladiata | Cgl |
| Cicer arietinum | Car |
| Cicer reticulatum | Cre |
| Cyamopsis tetragonoloba | Cte |
| Glycine max | Gma |
| Lablab purpureus | Lpu |
| Lathyrus cicera | Lci |
| Lathyrus ochrus | Loc |
| Lathyrus sativus | Lsa |
| Lens culinaris | Lcu |
| Lupinus albus | Lal |
| Lupinus angustifolius | Lan |
| Lupinus luteus | Llu |
| Lupinus mutabilis | Lmu |
| Macrotyloma uniflorum | Mun |
| Mucuna pruriens | Mpr |
| Phaseolus acutifolius | Pac |
| Phaseolus coccineus | Pco |
| Phaseolus lunatus | Plu |
| Phaseolus vulgaris | Pvu |
| Pisum humile | Phu |
| Pisum sativum | Psa |
| Psophocarpus tetragonolobus | Pte |
| Sphenostylis stenocarpa | Sst |
| Trigonella foenum-graecum | Tfo |
| Vicia ervilia | Ver |
| Vicia faba | Vfa |
| Vicia monantha | Vmo |
| Vicia narbonensis | Vna |
| Vicia sativa | Vsa |
| Vigna aconitifolia | Vac |
| Vigna angularis | Van |
| Vigna mungo | Vmu |
| Vigna radiata | Vra |
| Vigna umbelatta | Vum |
| Vigna unguiculata | Vun |

Supplementary Table S2 : Pearson correlation between 6 agroecosystem properties. See Table 2 for abbreviations.

|  | Grain yield | Biomass | LAI | Ndfa | Yield reduction |
| --- | --- | --- | --- | --- | --- |
| Grain yield |  |  |  |  |  |
| Biomass | 0.69*** |  |  |  |  |
| LAI | 0.02 | 0.35 |  |  |  |
| Ndfa | 0.13 | 0.22 | 0.33 |  |  |
| Yield reduction | 0.27 | 0.09 | -0.15 | 0.04 |  |
| WUE | 0.07 | 0.27 | 0.35 | 0.09 | -0.07 |

p < .001***

Supplementary Table S3 : Pearson’s correlation coefficients between 15 functional traits. See Table 1 for abbreviations.

|  | DM | DF | Seed_size | lflt_nb | lflt_lth | lflt_wth | PH | TSW | LNC | Seed_oil | Seed_prot | LA | SLA S/R |
| --- | --- | --- | --- | --- | --- | --- | --- | --- | --- | --- | --- | --- | --- |
| DM |  |  |  |  |  |  |  |  |  |  |  |  |  |
| DF | 0.7*** |  |  |  |  |  |  |  |  |  |  |  |  |
| Seed_size | 0.16 | -0.11 |  |  |  |  |  |  |  |  |  |  |  |
| lflt_nb | 0.25 | 0.23 | -0.34 |  |  |  |  |  |  |  |  |  |  |
| lflt_lth | -0.15 | -0.25 | 0.5*** | -0.81*** |  |  |  |  |  |  |  |  |  |
| lflt_wth | -0.23 | -0.34* | 0.43*** | -0.73*** | 0.9*** |  |  |  |  |  |  |  |  |
| PH | 0.25 | 0.11 | 0.46** | -0.45** | 0.6*** | 0.55*** |  |  |  |  |  |  |  |
| TSW | 0.14 | -0.12 | 0.87*** | -0.35 | 0.51*** | 0.46** | 0.47** |  |  |  |  |  |  |
| LNC | -0.12 | -0.04 | 0.1 | -0.18 | 0.09 | -0.01 | 0.22 | 0.17 |  |  |  |  |  |
| Seed_oil | 0.13 | -0.16 | 0.37* | 0.09 | 0.06 | 0.06 | 0.03 | 0.34* | -0.12 |  |  |  |  |
| Seed_prot | 0.26 | 0.13 | -0.18 | 0.26 | -0.24 | -0.32* | -0.15 | -0.19 | 0.21 | 0.45** |  |  |  |
| LA | -0.1 | -0.13 | 0.42** | -0.56*** | 0.61*** | 0.55** | 0.44** | 0.46** | 0.26 | 0.43** | 0.04 |  |  |
| SLA | -0.22 | -0.2 | 0.36* | -0.22 | 0.24 | 0.13 | -0.05 | 0.29 | 0.24 | -0.12 | -0.12 | 0.05 |  |
| S/R | 0.03 | 0.08 | 0.09 | -0.33* | 0.5*** | 0.52** | 0.29 | 0 | -0.4** | -0.09 | -0.36* | 0.1 | -0.15 |
| HI | 0.03 | 0 | 0.18 | 0.26 | -0.31* | -0.25 | -0.26 | 0.14 | -0.09 | 0.14 | 0 | -0.17 | 0.25 -0.24 |

p < .05*, p<0.01**, p<0.001***

Supplementary table S4 : Complete list of references used to build the database.

| Authors | Title | Date | Journal/publisher | Web |
| --- | --- | --- | --- | --- |
| Bolaji U. Olayinka, Emmanuel O. Etejere | Growth analysis and yield of two varieties of groundnut (Arachis hypogaea L.) as influenced by different weed control methods | 2015 | Indian J Plant Physiol. | <https://www.ncbi.nlm.nih.gov/pmc/articles/PMC4439525/> |
| Rainer Gross | El cultivo y la utilización del tarwi: Lupinus mutabilis Sweet | 1982 | FAO | <https://books.google.fr/books/about/El_cultivo_y_la_utilizaci%C3%B3n_del_tarwi.html?id=uVYzAAAACAAJ&redir_esc=y> |
| Wilson Urrutia Gutiérrez | Determinación de parámetros óptimos de extracción alcalina para la obtención de aislado proteico a partir de tarwi (lupinus mutabilis) | 2012 | UNAMBA | <http://repositorio.unamba.edu.pe/handle/UNAMBA/311> |
| James A. Duke | Handbook of Energy Crops. | 1983 | New Crop Resource Online Program | <https://hort.purdue.edu/newcrop/duke_energy/Arachis_hypogaea.html> |
| K.J. Boote | Growth Stages of Peanut (Arachis hypogea L.) | 1962 | Peanut science | <http://www.peanutscience.com/doi/pdf/10.3146/i0095-3679-9-1-11> |
| K.F. El-Sahhar,Kh. S. Emara, W.A. Ali, | Comparative Systematic Studies of Astragalus in flora of Arab Republic of Egypt and Syrian Arab Republic | 2013 | RJABS | <http://www.aensiweb.net/AENSIWEB/rjabs/rjabs/2013/271-286.pdf> |
| Aziza Zoghlami, Mongi Zouaghi | Morphological variation in Astragalus hamosus L. and Coronilla scorpioides L. populations of Tunisia | 2003 | Euphytica | <https://eurekamag.com/pdf/004/004241941.pdf> |
| Jaime Prohens, Isabel Andújar, Santiago Vilanova, Mariola Plazas, Pietro Gramazio, Rafael Prohens, Francisco J. Herraiz, Antonio M. De Ron | Swedish coffee (Astragalus boeticus L.), a neglected coffee substitute with a past and a potential future | 2014 | Genet. Resour. Crop Evol. |  |
| Nasir El Bassam | Handbook of bioenergy crops : a complete reference to species, development and applications | 2010 |  | https://books.google.fr/books?id=zRW3_QwQhTIC&pg=PA199&lpg=PA199&dq=Arachis+hypogaea+biomasse&source=bl&ots=52gznMjMHl&sig=4AaxjSE8_ykLT9DhC9LiJfvQHws&hl=fr&sa=X&ved=0ahUKEwimwIeY1JvcAhVMyqQKHeVzCM84ChDoAQg9MAM#v=onepage&q=Arachis hypogaea biomasse&f=false |
| Hector Valenzuela, Jody Smith | Green Manure Crops: Lablab | 2002 |  | <https://www.ctahr.hawaii.edu/oc/freepubs/pdf/GreenManureCrops/lablab.pdf> |
| M.J. Du Plessis, D. Fourie, A.J. Liebenberg, M.M. Liebenberg, C.J. Van Zyl | Dry bean | 2001 | Department of Agriculture, Forestry and Fisheries | <https://www.nda.agric.za/docs/drybean/drybean.pdf> |
| J. Smartt | The groundnut crop; a scientific basis for improvment. | 2012 | Springer Science & Business Media | <https://books.google.fr/books?hl=fr&lr=&id=kIP1CAAAQBAJ&oi=fnd&pg=PR15&ots=AL639fWp2K&sig=i8ZzxC31vQwPcRHT-qrjMrTqzZU&redir_esc=y#v=onepage&q&f=false> |
| Tendai P. Chibarabada, Albert T. Modi, Tafadzwanashe Mabhaudhi | Expounding the value of grain legumes in the semi- and arid tropics | 2017 | Sustainability (Switzerland) | <http://www.mdpi.com/2071-1050/9/1/60> |
| Carlos German Muñoz-Perea, Richard G. Allen, Dale T. Westermann, James L. Wright, Shree P. Singh | Water use efficiency among dry bean landraces and cultivars in drought-stressed and non-stressed environments | 2007 | Euphytica | <https://link.springer.com/article/10.1007%2Fs10681-006-9340-z> |
| Gn Patel, Pt Patel, Ph Patel | Yield, water use efficiency and moisture extraction pattern of summer groundnut as influenced by irrigation schedules, sulfur levels and sources | 2008 | Journal of SAT Agricultural Research | <http://ejournal.icrisat.org/Volume6/Groundnut/GN_Patel.pdf> |
| H. Zhang, M. Pala,T. Oweis, H. Harris | Water use and water-use efficiency of chickpea and lentil in a Mediterranean environment | 2000 | Australian Journal of Agricultural Research | <http://www.publish.csiro.au/?paper=AR99059> |
| Sunday Ewele Obalum, Charles Arizechukwu Igwe, Martin Eze Obi, Toshiyuki Wakatsuki | Water use and grain yield response of rainfed soybean to tillage-mulch practices in southeastern Nigeria | 2011 | Scientia Agricola | <http://www.scielo.br/scielo.php?script=sci_arttext&pid=S0103-90162011000500007&lng=en&tlng=en> |
| Y. A. Abayomi, T. V. Ajibade, O. F. Sammuel, B. F. Sa'adudeen | Growth and yield responses of cowpea (Vigna unguiculata (L.) Walp) genotypes to nitrogen fertilizer (NPK) application in the Southern Guinea Savanna zone of Nigeria | 2008 | Asian Journal of Plant Sciences | <http://www.docsdrive.com/pdfs/ansinet/ajps/2008/170-176.pdf> |
| L Vimalendran, K R Latha | Yield, water use and water use efficiency of pigeonpea [Cajanus cajan (L.) Millsp.] under drip fertigation system | 2014 | J. Appl. Nat. Sci |  |
| K. H.M. Siddique, K. L. Regan, D. Tennant, B. D. Thomson | Water use and water use efficiency of cool season grain legumes in low rainfall Mediterranean-type environments | 2001 | European Journal of Agronomy | <https://www.sciencedirect.com/science/article/pii/S116103010100106X> |
| P. Geil, J. Anderson | Nutrition and health implications of dry beans: a review. | 1994 | Journal of the American College of Nutrition | http://www.ncbi.nlm.nih.gov/pubmed/7706585 |
| V. N. Atasie, T. F. Akinhanmi, C. C. Ojiodu | Proximate analysis and physico-chemical properties of groundnut (Arachis hypogaea L.) | 2009 | Pakistan Journal of Nutrition | http://www.scialert.net/abstract/?doi=pjn.2009.194.197 |
| Patti Bazel Geil, James W Anderson | Nutritional quality of important food legumes | 2006 | Food Chemistry | <https://www.sciencedirect.com/science/article/pii/S0308814605003687?via%3Dihub> |
| Keshun Liu | Soybeans : Chemistry and Nutritional Value of Soybean Components | 1997 | Springer US | <http://link.springer.com/10.1007/978-1-4615-1763-4_2> |
| R. K. Deka, C. R. Sarkar | Nutrient composition and antinutritional factors of Dolichos lablab L. seeds | 1990 | Food Chemistry | <https://www.sciencedirect.com/science/article/pii/030881469090180C> |
| K.E. Akande, M.M. Abubakar, T.A. Adegbola, S.E. Bogoro, U.D. Doma | Chemical Evaluation of the Nutritive Quality of Pigeon Pea [Cajanus cajan (L.) Millsp.] | 2010 | International Journal of Poultry Science | https://www.researchgate.net/publication/49965141_Chemical_Evaluation_of_the_Nutritive_Quality_of_Pigeon_Pea_Cajanus_cajan_L_Millsp |
| Katell Crépon, Pascal Marget, Corinne Peyronnet, Benoit Carrouée, Paolo Arese, Gérard Duc | Nutritional value of faba bean (Vicia faba L.) seeds for feed and food | 2010 | Field Crops Research | <https://www.sciencedirect.com/science/article/pii/S0378429009002512> |
| Vladimír Vecerek, Suchý Pavel, Eva Straková, Miroslav Machácek, | Nutritive Composition of Seeds of The Lupin Varasundharosoth Arieties Registered in The Czech Republic | 2008 | International Lupin Association | <https://www.cabdirect.org/cabdirect/abstract/20103116731> |
| B.R. Ntare, | Arachis hypogaea L. | 2007 | PROTA | <http://www.prota4u.org/search.asp> |
| L.J.G. Van Der Maesen | Cajanus cajan (L.) Millsp. | 2006 | PROTA | <http://www.prota4u.org/search.asp> |
| Y.K. Chee, J.B. Hacker, L. Ramirez, C.P. Chen | Canavalia ensiformis | 2016 | PROTA | https://uses.plantnet-project.org/en/Canavalia_ensiformis_(PROSEA) |
| C.H. Bosch | Canavalia gladiata (Jacq.) DC. | 2004 | PROTA | <http://www.prota4u.org/search.asp> |
| G. Bejiga, L.J.G. Van Der Maesen | Cicer arietinum L. | 2006 | PROTA | <http://www.prota4u.org/search.asp> |
| Efloras | Cyamopsis tetragonoloba (Linn.). | 2008 | Missouri Botanical Garden, St. Louis, MO & Harvard University Herbaria, Cambridge, MA | [http://www.efloras.org](http://www.efloras.org/) |
| L.J. Wong, C. Parmar | Cyamopsis tetragonoloba | 2016 | PROSEA | https://uses.plantnet-project.org/en/Cyamopsis_tetragonoloba_(PROSEA) |
| K.E. Giller, K.E. Dashiell | Glycine max (L.) Merr. | 2007 | PROTA | <https://www.prota4u.org/database/protav8.asp?h=M26&t=Glycine_max&p=Glycine+max#MajorReferences> |
| A.A. Adebisi, C.H. Bosch | Lablab purpureus (L.) Sweet. | 2004 | PROTA | <https://www.prota4u.org/database/protav8.asp?h=M26&t=Lablab_purpureus&p=Lablab+purpureus#MajorReferences> |
| Wesley J. Everman, Scott B. Clewis, Walter E. Thomas, Ian C. Burke, John W. Wilcut | Critical Period of Weed Interference in Peanut | 2008 | Weed Technology | http://www.bioone.org/doi/abs/10.1614/WT-07-052.1 |
| G. Mohammadi, A. Javanshir, F. R. Khooie, S. A, Mohammadi, S. Zehtab Salmasi | Critical period of weed interference in chickpea | 2005 | Weed Research | https://onlinelibrary.wiley.com/doi/pdf/10.1111/j.1365-3180.2004.00431.x |
| A. J. Kerkhoff, W. F. Fagan, J. J. Elser, B. J. Enquist. | Phylogenetic and growth form variation in the scaling of nitrogen and phosphorus in the seed plants | 2006 | American Naturalist : In Try Database | https://www.journals.uchicago.edu/doi/10.1086/507879 |
| Wenxuan Han, Yahan Chen, Fang-Jie Zhao, Luying Tang, Rongfeng Jiang, Fusuo Zhang | Floral, climatic and soil pH controls on leaf ash content in China’s terrestrial plants | 2012 | Global Ecology and Biogeography | http://doi.wiley.com/10.1111/j.1466-8238.2011.00677.x |
| Royal Botanic Gardens Kew | Seed Information Database (SID) | 2008 |  | <http://data.kew.org/sid/> |
| Jarunee Pilumwong, Chuckree Senthong, Sombat Srichuwong, Keith T. Ingram, | Effects of temperature and elevated CO2 on shoot and root growth of peanut (Arachis hypogaea L.) grown in controlled environment chambers | 2007 | ScienceAsia | http://scienceasia.org/2007.33.n1/v33_079_087.pdf |
| L. Krishnamurthy, V. Vadez, M. Jyotsna Devi, R. Serraj, S.N. Nigam, M.S. Sheshshayee, S. Chandra, R. Aruna, | Variation in transpiration efficiency and its related traits in a groundnut (Arachis hypogaea L.) mapping population | 2007 | Field Crops Research | https://www.sciencedirect.com/science/article/pii/S0378429007000986 |
| P. Songsri,S. Jogloy, C. C. Holbrook, T. Kesmala, N. Vorasoot, C. Akkasaeng, A. Patanothai | Association of root, specific leaf area and SPAD chlorophyll meter reading to water use efficiency of peanut under different available soil water | 2009 | Agricultural Water Management | <https://www.sciencedirect.com/science/article/pii/S0378377408002825> |
| P. Q. Craufurd, P. V. Vara Prasad, R. J. Summerfield, | Dry matter production and rate of change of harvest index at high temperature in peanut | 2002 | Crop Science | https://www.researchgate.net/publication/11601897_Dry_Matter_Production_and_Rate_of_Change_of_Harvest_Index_at_High_Temperature_in_Peanut |
| M. Kleyer, R. M. Bekker, I. C. Knevel, J. P. Bakker, K. Thompson, M. Sonnenschein, P. Poschlod, J. M. Van Groenendael, L. Klimes, J. Klimesova, S. Klotz, G. M. Rusch, Hermy, M. , D. Adriaens, G. Boedeltje, B. Bossuyt, A. Dannemann, P. Endels, L. Götzenberger, J. G. Hodgson, A.-K. Jackel, I. Kühn, D. Kunzmann, W. A. Ozinga, C. Römermann, M. Stadler, J. Schlegelmilch, H. J. Steendam, O. Tackenberg, B. Wilmann, J. H. C. Cornelissen, O. Eriksson, E. Garnier, B. Peco. | The LEDA Traitbase: a database of life-history traits of the Northwest European flora | 2008 | Journal of Ecology | http://doi.wiley.com/10.1111/j.1365-2745.2008.01430.x |
| Kattge, J. Et Al. | TRY - a global database of plant traits | 2011 | Global Change Biology | http://doi.wiley.com/10.1111/j.1365-2486.2011.02451.x |
| I. Colin Prentice, Tingting Meng, Han Wang, Sandy P. Harrison, Jian Ni, Guohong Wang | Evidence of a universal scaling relationship for leaf CO2 drawdown along an aridity gradient | 2011 | New Phytologist | <http://doi.wiley.com/10.1111/j.1469-8137.2010.03579.x> |
| Benjamin Blonder, Vanessa Buzzard, Irena Simova, Lindsey Sloat, Brad Boyle, Rebecca Lipson, Brianna Aguilar-Beaucage, Angelina Andrade, Benjamin Barber, Chris Barnes, Dharma Bushey, Paulina Cartagena, Max Chaney, Karina Contreras, Mandarava Cox, Maya Cueto, Cannon Curtis, Mariah Fisher, Lindsey Furst, Jessica Gallegos, Ruby Hall, Amelia Hauschild, Alex Jerez, Nadja Jones, Aaron Klucas, Anita Kono, Mary Lamb, Jacob David Ruiz Matthai, Colten Mcintyre, Joshua Mckenna, Nicholas Mosier, Maya Navabi, Alex Ochoa, Liam Pace, Ryland Plassmann, Rachel Richter, Ben Russakoff, Holden St Aubyn, Ryan Stagg, Marley Sterner, Emily Stewart, Ting Ting Thompson, Jake Thornton, Parker J. Trujillo, Trevor J. Volpe, Brian J. Enquist | The leaf-area shrinkage effect can bias paleoclimate and ecology research | 2012 | American Journal of Botany | https://www.researchgate.net/publication/232969813 The |
| V. Heuzé ,H. Thiollet , G. Tran , R. Delagarde , D. Bastianelli , F. Lebas | *Pigeon pea (Cajanus cajan) forage* | 2017 | Feedipedia | <https://www.feedipedia.org/node/22444> |
| Florence Tardy, Delphine Moreau, Marc Dorel, Gaëlle Damour | Trait-based characterisation of cover plants’ light competition strategies for weed control in banana cropping systems in the French West Indies | 2015 | European Journal of Agronomy | <https://www.sciencedirect.com/science/article/pii/S1161030115300174> |
| V Tomar S Singh G Tripathi S | Crop weed competitions in arhar (Cajanus cajan L.) under northern west plain zone | 2003 | Indian Journal of Weed Science | http://www.indianjournals.com/ijor.aspx?target=ijor:ijws&volume=35&issue=3and4&article=014 |
| A. R. Sheldrake, A. Narayanan | Growth, development and nutrient uptake in pigeonpeas ( Cajanus cajan) | 1979 | The Journal of Agricultural Science | http://www.journals.cambridge.org/abstract_S0021859600053752 |
| L Vimalendran, K R Latha, | Yield, water use and water use efficiency of pigeonpea [Cajanus cajan (L.) Millsp.] under drip fertigation system | 2014 | J. Appl. Nat. Sci | https://journals.ansfoundation.org/index.php/jans/article/view/482 |
| M. Vanaja, M. Maheswari, P. Sathish, P. Vagheera, N. Jyothi Lakshmi, G. Vijay Kumar, S. K. Yadav, Abdul Razzaq, Jainender Singh, B. Sarkar | Genotypic variability in physiological, biomass and yield response to drought stress in pigeonpea | 2015 | Physiol Mol Biol Plants. | <http://www.ncbi.nlm.nih.gov/pubmed/26600680> |
| Remmy Raphael Mwakimbwala | Comparative improvement in soil nitrogen by five leguminous cover crops at Uyole agricultural research institute, Mbeya, Tanzania | 2015 |  | http://www.suaire.suanet.ac.tz:8080/xmlui/bitstream/handle/123456789/1219/REMMY%20RAPHAEL%20MWAKIMBWALA.pdf?sequence=1&isAllowed=y#%5B40%2C%7B%22name%22%3A%22XYZ%22%7D%2C99%2C725%2C0%5 |
| K. Balakrishnan, N. Natarajaratnam, C. Rajendran | Critical Leaf Area Index in Pigeonpea | 2008 | Journal of Agronomy and Crop Science | https://www.researchgate.net/publication/230535821_Critical_Leaf_Area_Index_in_Pigeonpea |
| F. Ganry, H.G. Diem, J. Wey, Y.R. Dommergues | Inoculation with Glomus mosseae improves Nz fixation by field-grown soybeans | 1985 | Biol Fert Soils | https://link.springer.com/article/10.1007/BF00710966#citeas |
| H A Gibson, L B Dreyfus, R Y Dommergues, | Nitrogen fixation by legumes in the tropics | 1982 | Microbiology of Tropical Soils and Plant Productivity | http://horizon.documentation.ird.fr/exl-doc/pleins_textes/divers16-03/17372.pdf |
| Ranjeet Patel, Singh Rkr, Varun Tyagi, Mallesha, Raju Ps | Nutritional evaluation of Canavalia ensiformis (Jack bean ) cultivated in North East region of India | 2016 | International Journal of Botany Studies | [www.botanyjournals.com/download/63/1-6-13-267.pdf](http://www.botanyjournals.com/download/63/1-6-13-267.pdf) |
| Suriyan Cha-Um, Charlie B. Batin, Thapanee Samphumphung, Chalermpol Kidmanee | Physio-morphological changes of cowpea (vigna unguiculata walp.) And jack bean (canavalia ensiformis (L.) DC.) In responses to soil salinity | 2013 | Australian Journal of Crop Science | https://www.researchgate.net/publication/286073591_Physio-morphological_changes_of_cowpea_vigna_unguiculata_walp_And_jack_bean_canavalia_ensiformis_L_DC_In_responses_to_soil_salinity |
| Virupax C. Baligar, Marshall Elson, Zhenli L. He, Yuncong Li, Arlicelio De Q. Paiva, Dario Ahnert, Alex-Alan F. Almeida And Nand K. Fageria | Ambient and Elevated Carbon Dioxide on Growth, Physiological and Nutrient Uptake Parameters of Perennial Leguminous Cover Crops under Low Light Intensities | 2015 | International Journal of Plant & Soil Science | http://www.journalrepository.org/media/journals/IJPSS_24/2017/Apr/Baligar1542017IJPSS32790_1.pdf |
| C. D. J. Kessler | An Agronomic Evaluation of Jackbean (Canavalia Ensiformis) in Yucatan, Mexico. I. Plant Density | 1990 | Experimental Agriculture | http://www.journals.cambridge.org/abstract_S0014479700015350 |
| Muhammad Akhsan Akib, Kahar Mustari, Tutik Kuswinanti, Syatrianty Andi Syaiful | The effect of application acaulospora sp on the root growth of canavalia ensiformis l at nickel post-mine land | 2018 | Pak. J. Biotechnol | http://www.pjbt.org/uploads/2018/Vol-5/PJBT-VOL-15-NO-2-OF-YEAR-2018 (46).pdf |
| Banyong Toomsan, Viriya Limpinuntana, Sanun Jogloy, Aran Patanothai, Prabhakar Pathak, Suhas P Wani, Kl Sahrawat | Role of Legumes in Improving Soil Fertility and Increasing Crop Productivity in Northeast Thailand | 2012 | Community Watershed Management for Sustainable Intensification in Northeast Thailand | http://oar.icrisat.org/6538/1/Thailand_Report_Ch3.pdf |
| V. Vadivel, K. Janardhanan, K. Vijayakumari | Diversity in swordbean (Canavalia gladiata (Jacq.) DC.) collected from Tamil Nadu, India | 1998 | Genet. Resour. Crop Evol. | [https://sci-hub.tw/https://link.springer.com/article/10.1023/A:1008638101502](https://sci-hub.tw/https:/link.springer.com/article/10.1023/A:1008638101502) |
| Kamel Ben Mbarek, Abdelhamid Boujelben, Mohsen Boubaker, Chérif Hannachi | Criblage et performances agronomiques de 45 génotypes de pois chiche (Cicer arietinum L.) soumis à un régime hydrique limité | 2009 | Biotechnol. Agron. Soc. Environ. | <https://popups.uliege.be/1780-4507/index.php?id=4512> |
| Piara Singh, S.M. Virmani | Modeling growth and yield of chickpea (Cicer arietinum L.) | 1996 | Field Crops Research | https://www.sciencedirect.com/science/article/pii/0378429095000852 |
| Ramamoorthy Purushothaman, Lakshmanan Krishnamurthy, Hari Deo Upadhyaya, Vincent Vadez, Rajeev Kumar Varshney | Shoot traits and their relevance in terminal drought tolerance of chickpea (Cicer arietinum L.) | 2016 | Field Crops Research | https://www.sciencedirect.com/science/article/pii/S037842901630226X |
| Ranjeet Kour, B.C. Sharma, Anil Kumar, Paramjeet Kour, Brij Nandan | Study of physiological growth indices of mustard in chickpea (cicer arietinum)+ mustard (brassica juncea) intercropping system under different weed management practices | 2016 | Indian Journal of Agricultural Research | https://pdfs.semanticscholar.org/1f5b/2c4d25b60be640cbe7e57b961e7e79b04947.pdf |
| M. Ashraf, A. Waheed | Screening chick-pea (Cicer arietinum L.) for salt tolerance | 1992 | Der Tropenlandwirt, Zeitschrift fur die landwirtschaft in den Tropen und Subtropen | https://www.jarts.info/index.php/tropenlandwirt/article/viewFile/987/292 |
| L Krishnamurthy, C. Johansen, O. Ito | Genotypic Variation in Root System Development and Its Implications for Drought Resistance in Chickpea | 1996 | Dynamics of Roots and Nitrogen in Cropping Systems of the Semi-Arid Tropics |  |
| Yinglong Chen, Michel Edmond Ghanem, Kadambot Hm Siddique, | Characterising root trait variability in chickpea (Cicer arietinum L.) germplasm | 2017 | Journal of Experimental Botany | http://www.ncbi.nlm.nih.gov/pubmed/28338728 |
| Muhammad Arshad, A. Bakhsh, Abdul Ghafoor | Path Coefficient Analysis In Chickpea (Cicer Arietinum L.) Under Rainfed Conditions | 2004 | Pak. J. Bot. | https://www.researchgate.net/profile/Muhammad_Arshad56/publication/266456595_Path_coefficient_analysis_in_chickpea_Cicer_arietinum_L_under_rainfed_conditions/links/54fe9b900cf2eaf210b35ec4/Path-coefficient-analysis-in-chickpea-Cicer-arietinum-L-under-rainfed-conditions.pdf |
| D.F. Beech, H. Stutzel, D.A. Charles-Edwards | Yield determinants of Guar (Cyamopsis tetragonoloba): 1. Grain yield and pod number | 1989 | Field Crops Research | https://www.sciencedirect.com/science/article/pii/0378429089900385 |
| A. R. Mubarak, N. O. Salih And A. A. Hassabo | Fate of 15N-labeled urea under a guar-wheat rotation as influenced by crop residue incorporation in a semi-arid Vertisol. | 2016 | Tropical Agriculture | https://journals.sta.uwi.edu/ta/index.asp?action=viewPastAbstract&articleId=899&issueId=135 |
| S. L. Yadav, M. K. Kaushik, S. L. Mundra | Effect of Weed Control Practices on Weed Dry Weight, Nutrient Uptake and Yield of Clusterbean[Cyamopsis tetragonoloba (L.) Taub.] under Rainfed Condition | 2011 | Indian J. Weed Sci. | http://isws.org.in/IJWSn/File/2011_43_Issue-1&2_81-84.pdf |
| R. S. Yadav | Effects of Weed Removal in Clusterbean (Cyamopsis tetmgonoloba) under Different Rainfall Situations in an Arid Region | 1998 | J. Agronomy & Crop Science | https://onlinelibrary.wiley.com/doi/epdf/10.1111/j.1439-037X.1998.tb00419.x |
| B.S. Afria, N.S. Nathawat, M.L Yadav, | Effect of cycocel and saline irrigation of physiological attributes, yield and its components in different - varieties of guar ( cyamopsis tetragonoloba l . Taub .) | 1998 | Indian J. Plant Physiol. | http://inorderbox.com/siteimages/publication_file/4/ijpp-3-1-010.pdf |
| H. S. Talwar, M. L. Soni, R. K. Beniwal, N. D. Yadava, J. P. Singh, V. S. Rathore, Sunil Kumar, | Crop physiological approaches to evaluate drought-resistant traits in clusterbean (Cyamopsis tetragonoloba) | 2008 | Indian Journal of Agricultural Sciences | <https://www.academia.edu/21387082/Crop_physiological_approaches_for_drought_tolerance_in_clusterbean> |
| Samrath Lal Meena, M. Shamsudheen, Devi Dayal, | Productivity of clusterbean (Cyamopsis tetragonoloba) and sesame (Sesamum indicum) intercropping system under different row ratio and nutrient management in arid region | 2009 | Indian Journal of Agricultural Sciences | http://epubs.icar.org.in/ejournal/index.php/IJAgS/issue/view/42 |
| V. S. Rathore, J. P. Singh, M. L. Soni, R. K. Beniwal, | Effect of nutrient management on growth, productivity and nutrient uptake of rainfed clusterbean (Cyamopsis tetragonoloba) in arid region | 2007 | Indian Journal of Agricultural Sciences | https://www.researchgate.net/publication/262180032_Effect_of_nutrient_management_on_growth_productivity_and_nutrient_uptake_of_rainfed_clusterbean_Cyamopsis_tetrwgonolobain_arid_region |
| J. C. Tarafdar, A. V. Rao, Praveen Kumar, | Role of phosphatase-producing fungi on the growth and nutrition of clusterbean (Cyamopsis tetragonoloba (L.) Taub.) | 1995 | Journal of Arid Environments | www.sciencedirect.com/science/article/pii/S0140196305801120 |
| Ayda Krisnawati, M. Muchlish Adie, | Variability of Biomass and Harvest Index from Several Soybean Genotypes as Renewable Energy Source | 2015 | Energy Procedia | http://dx.doi.org/10.1016/j.egypro.2015.01.023 |
| Y. Gan, I. Stulen, H. Van Keulen, P.J.C. Kuiper | Physiological response of soybean genotypes to plant density | 2002 | Field Crops Research | <https://www.sciencedirect.com/science/article/pii/S037842900100212X> |
| Donald T. Krizek, Avner Carmi, Roman M. Mirecki, Freeman W. Snyder, James A. Bunce | Comparative effects of soil moisture stress and restricted root zone volume on morphogenetic and physiological responses of soybean [Glycine max (L.) Merr.] | 1985 | Journal of Experimental Botany | https://academic.oup.com/jxb/article-lookup/doi/10.1093/jxb/36.1.25 |
| K. M. Hati, K. G. Mandal, A. K. Misra, P. K. Ghosh, K. K. Bandyopadhyay, | Effect of inorganic fertilizer and farmyard manure on soil physical properties, root distribution, and water-use efficiency of soybean in Vertisols of central India | 2006 | Bioresource Technology | https://www.sciencedirect.com/science/article/pii/S0960852405004797 |
| Y Pushpa Reni, Y Koteswara Rao | Genetic variability in soybean [ glycine max ( l ) merrill ] | 2013 | International Journal of Plant, Animal and Environmental Sciences | http://www.ijpaes.com/admin/php/uploads/384_pdf.pdf |
| Christopher M. Sheahan | Plant guide for lablab (Lablab purpureus). | 2012 | USDA | http://www.ijpaes.com/admin/php/uploads/384_pdf.pdf |
| N Anandaraja, Sankri S K, N Sriram, R Venkatachalam | TNAU Agri Tech Portal : Content Design and Validation | 2013 | CSI Communications | https://www.researchgate.net/publication/258424385_TNAU_Agri_Tech_Portal_Content_Design_and_Validation |
| R. E. Hendricksen, D. J. Minson, | Growth, Canopy Structure and chemical Composition of Lablab purpureus CV. Rongai at Samford, S.E. Queensland | 1985 | Tropical Grasslands | <http://www.tropicalgrasslands.asn.au/Tropical%20Grasslands%20Journal%20archive/PDFs/Vol_19_1985/Vol_19_02_85_pp81_87.pdf> |
| Achmad Subagio | Characterization of hyacinth bean (Lablab purpureus (L.) sweet) seeds from Indonesia and their protein isolate | 2006 | Food Chemistry | https://www.sciencedirect.com/science/article/pii/S0308814605000324 |
| V.C. Baligar, N.K. Fageria, A. Paiva, A. Silveira, J.O. De Souza Jr.,E. Lucena, J.C. Faria, R. Cabral, A.W.V. Pomella, And J. Jorda Jr. | Chapter 5 : Light Intensity Effects on Growth and Nutrient-use Efficiency of Tropical Legume Cover Crops | 2008 | Springer | <https://www.researchgate.net/profile/Clive_Welham/publication/226493655_The_Role_of_Ecosystem-level_Models_in_the_Design_of_Agroforestry_Systems_for_Future_Environmental_Conditions_and_Social_Needs/links/543ff2ad0cf21227a11b9d33/The-Role-of-Ecosystem-level-Models-in-the-Design-of-Agroforestry-Systems-for-Future-Environmental-Conditions-and-Social-Needs.pdf#page=75> |
| J. Neal, W. Fulkerson, R. Hacker | Differences in water use efficiency among annual forages used by the dairy industry under optimum and deficit irrigation | 2011 | Agricultural Water Management | <https://www.sciencedirect.com/science/article/pii/S037837741000377X> |
| R.C.Muchow | Canopy development in grain legumes grown under different soil water regimes in a semi-arid tropical environment | 1985 | Field Crops Research | <https://www.sciencedirect.com/science/article/pii/S037837741000377X> |
| L. M. Mugwira, I. Haque | Screening forage and browse legumes germplasm to nutrient stress: II. Tolerance of Lablab purpureus L. to acidity and low phosphorus in two acid soils | 1993 | Jounal of Plant Nutrition | <https://www.tandfonline.com/doi/abs/10.1080/01904169309364513> |
| P.V. Vaijayanthi, S. Ramesh, M. Byre Gowda, A. Mohan Rao, C.M. Keerthi, G.A. Marry Reena | Genetic variability for morpho-metric traits in Dolichos bean (Lablab purpureus L. Sweet) germplasm | 2015 | Journal of Food Legume | https://www.researchgate.net/profile/Keerthi_C_M2/publication/292615644_Genetic_variability_for_morpho-metric_traits_in_Dolichos_bean_Lablab_purpureus_L_Sweet_germplasm/links/56b046d908ae9ea7c3adb7b7.pdf#page=9 |
| C.S. Praharaj, N. Kumar, Ummed Singh, S.S. Singh, Jagdish Singh | Transplanting in pigeonpea - A contingency measure for realizing higher productivity in Eastern Plains of India | 2015 | Journal of Food Legume | https://www.researchgate.net/profile/Keerthi_C_M2/publication/292615644_Genetic_variability_for_morpho-metric_traits_in_Dolichos_bean_Lablab_purpureus_L_Sweet_germplasm/links/56b046d908ae9ea7c3adb7b7.pdf#page=10 |
| Santhappan Paulraj, Murugesan Senthilkumar, Jagdish Singh | Relative field responsiveness and dependency of chickpea to combined inoculationof Mesorhizobium Ciceri, arbuscular mycorrhizal fungi, phosphobacteria and phosphorus under inceptisol | 2015 | Journal of Food Legume | https://www.researchgate.net/profile/Keerthi_C_M2/publication/292615644_Genetic_variability_for_morpho-metric_traits_in_Dolichos_bean_Lablab_purpureus_L_Sweet_germplasm/links/56b046d908ae9ea7c3adb7b7.pdf#page=11 |
| A.N. Sahane, R. D. Deotale, S.A. Mahale, Shanti R. Patil, P.P. Sawant | Influence of ethrel on quality parameters and yield of soybean | 2015 | Journal of Food Legume | <https://www.researchgate.net/profile/Keerthi_C_M2/publication/292615644_Genetic_variability_for_morpho-metric_traits_in_Dolichos_bean_Lablab_purpureus_L_Sweet_germplasm/links/56b046d908ae9ea7c3adb7b7.pdf#page=11> |
| Dhiman Mukherjee | Integrated nutrient management practices for enhancing blackgram (Vigna mungo L. Hepper) production under mid-hill situation in North Eastern Himalaya | 2015 | Journal of Food Legume | <https://www.researchgate.net/profile/Keerthi_C_M2/publication/292615644_Genetic_variability_for_morpho-metric_traits_in_Dolichos_bean_Lablab_purpureus_L_Sweet_germplasm/links/56b046d908ae9ea7c3adb7b7.pdf#page=11> |
| Pawar R. M., R. M. Prajapati, D. M. Sawant, A. H. Patil | Genetic divergence in Indian bean (Lablab purpureus L. Sweet) | 2013 | Electronic Journal of Plant Breeding | http://www.ejplantbreeding.org/index.php/EJPB/article/view/379 |
| A. M. Abd El Moneim, M. A. Khair, P. S. Cocks | Growth Analysis, Herbage and Seed Yield of Certain Forage Legume Species Under Rainfed Conditions | 1990 | J. Agronomy & Crop Science | https://onlinelibrary.wiley.com/doi/pdf/10.1111/j.1439-037X.1990.tb00783.x |
| M. Ratinam, A. M. Abd El Moneim, M. C. Saxena | Variations in Sugar Content and Dry Matter Distribution in Roots and their Associations with Frost Tolerance in Certain Forage Legume Species. | 1994 | J. Agronomy & Crop Science | https://onlinelibrary.wiley.com/doi/abs/10.1111/j.1439-037X.1994.tb00582.x |
| I. Vasilakoglou1, D. Vlachostergios, K. Dhima, A. Lithourgidis | Response of vetch, lentil, chickpea and red pea to pre- or post-emergence applied herbicides | 2013 | Spanish Journal of Agriculture Research | <http://revistas.inia.es/index.php/sjar/article/view/4083/1981> |
| Heuzé V., Tran G., Sauvant D., Bastianelli D., Lebas F. | *Lentil (Lens culinaris)*. | 2015 | [Feedipedia](https://www.feedipedia.org/node/284) | <https://www.feedipedia.org/node/284> |
| S. J. Whitehead, R. J. Summerfield, F. J. Muehlbauer, C. J. Coyne, R. H. Ellis, And T. R. Wheeler | Crop Improvement and the Accumulation and Partitioning of Biomass and Nitrogen in Lentil | 2000 | Crop Science | https://pubag.nal.usda.gov/pubag/downloadPDF.xhtml?id=23438&content=PDF |
| M. Andrews, B. A.Mckenzie, A. Joyce,M. E. Andrews, | The potential of lentil (Lens culinaris) as a grain legume crop in the UK: an assessment based on a crop growth model. | 2001 | Annals of Aplied Biology | <https://onlinelibrary.wiley.com/doi/abs/10.1111/j.1744-7348.2001.tb00142.x> |
| Kağan Kökten, Tolga Karaköy, Adil Bakoğlu, Mevlüt Akçura | Determination of salinity tolerance of some lentil (Lens culinaris M.) varieties | 2010 | Journal of Food, Agriculture & environment | https://www.researchgate.net/profile/Tolga_Karakoey/publication/266056325_Determination_of_salinity_tolerance_of_some_lentil_Lens_culinaris_M_varieties/links/563215d108ae506cea69103b/Determination-of-salinity-tolerance-of-some-lentil-Lens-culinaris-M-varieties.pdf |
| J.A. Duke | Handbook of legumes of world economic importance. | 1981 | Plenum Press, New York, USA | https://books.google.fr/books?id=XX3dBwAAQBAJ&printsec=frontcover&dq=Handbook+of+legumes+of+world+economic+importance+/+edited+by+James+A+Duke&hl=fr&sa=X&ved=0ahUKEwjMu7fQs97fAhVJz4UKHSsQCPQQ6AEIKTAA#v=onepage&q=Lupinus&f=false |
| Larson, K. J., Cassman, K. G., & Phillips, D. A. | Yield, Dinitrogen Fixation, and Aboveground Nitrogen Balance of Irrigated White Lupin in a Mediterranean Climate. | 1989 | Agronomy Journal | https://dl.sciencesocieties.org/publications/aj/abstracts/81/3/AJ0810030538 |
| Leonardo Sulas, Simone Canu, Luigi Ledda, Antonio Melchiorre Carroni, Mauro Salis | and nitrogen fixation potential from white lupine grown in rainfed Mediterranean environment | 2016 | Scientia Agricola | http://dx.doi.org/10.1590/0103-9016-2015-0299 |
| L. Lopez-Bellido, M. Fuentes, J.C.B. Lhamby, J.E. Castillo | Growth and yield of white lupin (Lupinus albus) under Mediterranean conditions: effect of sowing date | 1994 | Field Crops Research | https://www.sciencedirect.com/science/article/pii/0378429094900574 |
| Lingyun Cheng, Xiaoyan Tang, Carroll P. Vance, Philip J. White, Fusuo Zhang, Jianbo Shen | Interactions between light intensity and phosphorus nutrition affect the phosphate-mining capacity of white lupin (Lupinus albus L.) | 2014 | Journal of Experimental Botany | <https://doi.org/10.1093/jxb/eru135> |
| Ying Long Chen, Vanessa M. Dunbabin, Johannes A. Postma, Art J. Diggle, Jairo A. Palta, Jonathan P. Lynch, Kadambot H. M. Siddique, Zed Rengel | Phenotypic variability and modelling of root structure of wild Lupinus angustifolius genotypes | 2011 | Plant Soil | https://s3.amazonaws.com/academia.edu.documents/44781386/Phenotypic_variability_and_modelling_of_20160415-25617-1mku2ea.pdf?AWSAccessKeyId=AKIAIWOWYYGZ2Y53UL3A&Expires=1547024673&Signature=8CpQ4F0SISexGmNWCKj7HtRk2Pk%3D&response-content-disposition=inline%3B%20filename%3DPhenotypic_variability_and_modelling_of.pdf |
| Ean Greenwood, P Farrington, Jd Beresford | Characteristics of the canopy, root system and grain yield of a crop of Lupinus angustifolius cv. Unicrop | 1975 | Australian Journal of Agricultural Research | https://doi.org/10.1071/AR9750497 |
| P.F. White, A.D. Robson | Response of lupins (Lupinus angustifofis L.) and peas (Pisum sativum L.) to Fe deficiency induced by low concentratons of Fe in solution or by addition of HCO 3 | 1990 | Plant and Soil | https://link.springer.com/article/10.1007/BF00010742 |
| J.A. Palta, N.C. Turner, R.J. French, B.J. Buirchell | Physiological responses of lupin genotypes to terminal drought in a Mediterranean-type environment | 2007 | Annals of Aplied Biology | https://onlinelibrary.wiley.com/doi/epdf/10.1111/j.1744-7348.2007.00140.x |
| J. D. Berger, K. N. Adhikari, D. Wilkinson, B. J. Buirchell, M. W. Sweetingham | Ecogeography of the Old World lupins. 1. Ecotypic variation in yellow lupin (Lupinus luteus L.) | 2008 | Australian Journal of Agricultural Research | https://s3.amazonaws.com/academia.edu.documents/38514115/AJAR_Berger_et_al_YL_ecotypes.pdf?AWSAccessKeyId=AKIAIWOWYYGZ2Y53UL3A&Expires=1547029521&Signature=g2G%2Fx34Ctb8%2BX4E9p20%2Fqelolnk%3D&response-content-disposition=inline%3B%20filename%3DEcogeography_of_the_Old_World_lupins._1..pdf |
| A. Hardy, C. Huyghe, J. Papineau | Dry matter accumulation and partitioning, and seed yield in indeterminate Andean lupin (Lupinus mutabilis Sweet) | 1997 | Australian Journal of Agricultural Research | http://www.publish.csiro.au/cp/A96015 |
| Sven-Erik Jacobsen, Angel Mujica | Geographical distribution of the Andean lupin (Lupinus mutabilis Sweet) | 2008 | Plant Genetic Resources Newsletter, | http://www.bioversityinternational.org/fileadmin/_migrated/uploads/tx_news/NL_155_complete.pdf#page=5 |
| César E. Falconí | Lupinus mutabilis in Ecuador with special emphasis on anthracnose resistance | 2012 | Thesis, Wageningen University, Wageningen NL | https://library.wur.nl/WebQuery/wurpubs/fulltext/210228 |
| Dilip Kumar Majumdar | Pulse crop production : Principles and technologies | 2011 | PHI Learning Private Limited | https://books.google.fr/books?id=EUPKAmSgEgUC&pg=PA238&lpg=PA238&dq=macrotyloma+uniflorum+germination+hypogeal&source=bl&ots=Woselc5Iyc&sig=Vo8IVzJPzLynav7sEr4P7H4VMj4&hl=fr&sa=X&ved=0ahUKEwi5sb3ip6bcAhUDGewKHdpcBWcQ6AEIbjAM#v=onepage&q=macrotyloma%20uniflorum%20germination%20hypogeal&f=false |
| Stefan Hauser, Christian Nolte | Biomass production and N fixation of five Mucuna pruriens varieties and their effect on maize yields in the forest zone of Cameroon | 2002 | Journal of Plant Nutrition and Soil Science | https://onlinelibrary.wiley.com/doi/abs/10.1002/1522-2624%28200202%29165%3A1%3C101%3A%3AAID-JPLN101%3E3.0.CO%3B2-F |
| Perumal Siddhuraju, Karuppanan Vijayakumari, Karnam Janardhanan | Chemical Composition and Protein Quality of the Little-Known Legume, Velvet Bean (Mucuna pruriens (L.) DC.) | 1996 | Journal of Agricultural And Food Chemistry | https://pubs.acs.org/doi/full/10.1021/jf950776x |
| Waldemar Klassen, Maharanie Codallo, Inga A. Zasada, Aref A. Abdul-Baki | Characterization Of Velvetbean(Mucuna Pruriens) Lines For Cover Crop Use | 2006 | Proc. Fla. State Hort. Soc. | https://fshs.org/proceedings-o/2006-vol-119/FSHS%20119/p.258-262.pdf |
| F Mohamed, M Mohamed, N Schmitz-Eiberger, Keutgen, G Noga | Comparative Drought Postponing And Tolerance Potentials Of Two Tepary Bean Lines In Relation To Seed Yield | 2005 | African Crop Science Journal | https://www.researchgate.net/publication/27791972_Comparative_Drought_Postponing_And_Tolerance_Potentials_Of_Two_Tepary_Bean_Lines_In_Relation_To_Seed_Yield |
| Albert H. Markhart | Comparative Water Relations of Phaseolus vulgaris L. and Phaseolus acutifolius Gray | 1985 | Plant physiol. | <http://www.plantphysiol.org/content/plantphysiol/77/1/113.full.pdf> |
| Jeannette S. Bayuelo-Jiménez, Daniel G.Debouck,Jonathan P. Lynch | Growth, gas exchange, water relations, and ion composition of Phaseolus species grown under saline conditions | 2003 | Field Crops Research | <https://www.sciencedirect.com/science/article/pii/S037842900200179X> |
| I. Rao, S. Beebe, J. Polania, J. Ricaurte, C. Cajiao, R. Garcia, M. Rivera | Can tepary bean be a model for improvement of drought resistance in common bean? | 2013 | African Crop Science Journal | https://www.ajol.info/index.php/acsj/article/viewFile/95291/84638 |
| Harbans L. Bhardwaj, Muddappa Rangappa, And Anwar A. Hamama | Planting Date and Genotype Effects on Tepary Bean Productivity | 2002 | HortScience | http://hortsci.ashspublications.org/content/37/2/317.full.pdf |
| M. Gutierrez, J.A. Escalante-Estrada, M.T. Rodriguez-Gonzalez | Differences in Salt Tolerance Between Phaseolus vulgaris and Phaseolus coccineus Cultivars | 2009 | International Journal of Agricultural Research | http://docsdrive.com/pdfs/academicjournals/ijar/2009/270-278.pdf |
| M. L. P. Vargas-Vázquez; M. B. G. Irizar-Garza | Efecto del brasinoesteroide y densidad de población en la acumulación de biomasa y rendimiento de ayocote (Phaseolus Coccineus L.) | 2005 | Revista Chapingo Serie Horticultura | https://www.chapingo.mx/revistas/horticultura/contenido.php?id_articulo=103&id_revistas=1&id_revista_numero=8 |
| P. Gepts, D. G. Debouck | Origin, domestication, and evolution of the common bean (Phaseolus vulgaris L.) | 1991 | In: van Schoonhoven, A., Voysest, O. (eds.). Common beans: research for crop improvement. Commonwealth Agricultural Bureaux International, Wallingford, United Kingdom. | https://cgspace.cgiar.org/handle/10568/88263 |
| Nz Jovanovic, Jg Annandale | Crop growth model parameters of 19 summer vegetable cultivars for use in mechanistic irrigation scheduling models | 2000 | Water S.A | <https://www.researchgate.net/profile/John_Annandale/publication/267679732_Crop_growth_model_parameters_of_19_summer_vegetable_cultivars_for_use_in_mechanistic_irrigation_scheduling_models/links/54e301270cf296663798243c.pdf> |
| Marius Stefan, Neculai Munteanu,Vasile Stoleru, Marius Mihasan, Lucian Hritcu | Seed inoculation with plant growth promoting rhizobacteria enhances photosynthesis and yield of runner bean (Phaseolus coccineus L.) | 2013 | scientia horticulturae | https://www.sciencedirect.com/science/article/pii/S0304423812005833 |
| Raymond C. Dobert,Dale G. Blevins | Effect of seed size and plant growth on nodulation and nodule development in lima bean (Phaseolus lunatus L.) | 1993 | Plant and Soil | https://link.springer.com/article/10.1007/BF02185380 |
| Yacob Sirait, Wallace G. Pill, Walter E. Kee, Jr. | Lima Bean (Phaseolus lunatus L.)Response to Irrigation Regime and Plant Population Density | 1994 | HortScience | http://hortsci.ashspublications.org/content/29/2/71.full.pdf |
| Mark J. Vangessel, David W. Monks, And Quintin R. Johnson | Herbicides for Potential Use in Lima Bean (Phaseolus lunatus) Production | 2000 | Weed Technology | https://www.jstor.org/stable/3988832?seq=1#page_scan_tab_contents |
| L. H. Ziska, A. E. Hall, R. M. Hoover | Irrigation Management Methods for Reducing Water Use of Cowpea (Vigna unguiculata [L.] Walp.) and Lima Bean (Phaseolus lunatus L.) While Maintaining Seed Yield at Maximum Levels | 1985 | Irrig Sci | https://www.researchgate.net/profile/Anthony_Hall6/publication/227137566_Soil_and_plant_measurements_for_determining_when_to_irrigate_cowpeas_Vigna_unguiculata_L_Walp_grown_under_planned-water-deficits/links/56de15fa08aeb8b66f94a65d.pdf |
| Jess Annai Millar | Effect of Mycorrhizal Colonization and Light Limitation on Growth and Reproduction of Lima Bean (Phaseolus lunatus L.) | 2014 | University Honors Theses | https://pdxscholar.library.pdx.edu/cgi/viewcontent.cgi?article=1038&context=honorstheses |
| B.T. Scully, D.H. Wallace | Variation in and Relationship of Biomass, Growth Rate, Harvest Index, and Phenology to Yield of Common Bean | 1990 | J. Amer. Soc. Hort. Sci. | <http://journal.ashspublications.org/content/115/2/218.full.pdf> |
| Marcel G. Costa França, Anh Thu Pham Thi, Carlos Pimentel, Roberto O. Pereyra Rossiello, Yasmine Zuily-Fodil, Daniel Laffray | Differences in growth and water relations among Phaseolus vulgaris cultivars in response to induced drought stress | 2000 | Environmental and Experimental Botany | <https://www.sciencedirect.com/science/article/pii/S009884729900060X> |
| R. Wakrim, S. Wahbi, H. Tahi, B. Aganchich, R. Serraj | Comparative effects of partial root drying (PRD) and regulated deficit irrigation (RDI) on water relations and water use efficiency in common bean ( Phaseolus vulgaris L.) | 2005 | Agriculture, Ecosystems and Environment | https://ac.els-cdn.com/S0167880904003056/1-s2.0-S0167880904003056-main.pdf?_tid=ea05a0f9-3d6a-4d03-adaa-57dc201f055c&acdnat=1547132787_f754b7a673b12f717a328635374eaf5a |
| T. Boutraa, F. E. Sanders | Influence of Water Stress on Grain Yield and Vegetative Growth of Two Cultivars of Bean (Phaseolus vulgaris L.) | 2001 | Journal of Agronomy and Crop Science | https://onlinelibrary.wiley.com/doi/full/10.1046/j.1439-037X.2001.00525.x |
| Irena Rapčan, Gordana Bukvić, Sonja Grljušić, Tihana Teklić, Mladen Jurišić, | Field pea (pisum sativum l.) Biomass production depended on seed age and agroecological growth conditions | 2006 | [Poljoprivreda](https://hrcak.srce.hr/poljoprivreda) | https://hrcak.srce.hr/index.php?id_clanak_jezik=11572&show=clanak |
| E.S. Jensen | Seasonal patterns of growth and nitrogen fixation in field-grown pea. | 1987 | Plant and Soil | https://link.springer.com/article/10.1007/BF02371027 |
| Ries De Visser, Hans Lambers | Growth and the efficiency of root respiration of Pisum sativum as dependent on the source of nitrogen | 1983 | Physiol. Plant | https://onlinelibrary.wiley.com/doi/full/10.1111/j.1399-3054.1983.tb05739.x |
| H. A. Mooney, K. Fichtner, E.-D. Schulze | Growth, photosynthesis and storage of carbohydrates and nitrogen in Phaseolus lunatus in relation to resource availability | 1995 | Oecologia | <https://link.springer.com/article/10.1007/BF00365557> |
| Hossein Ghamari, Goudarz Ahmadvand | Growth Analysis of Dry Bean (Phaseolus vulgaris L.) in Different Weed Interference Situations | 2013 | Not. Sci. Biol. | https://notulaebiologicae.ro/index.php/nsb/article/view/9052/8529 |
| M. Ngouajio, J. Foko And D. Fouejio | The critical period of weed control in common bean (Phaseolus vulgaris L.) in Cameroon | 1997 | Crop Protection | https://ac.els-cdn.com/S0261219496000853/1-s2.0-S0261219496000853-main.pdf?_tid=a16d55e5-98ec-446c-8ae7-667371cd2b39&acdnat=1547137391_4de43e5a964b51010c7eb546e36c4129 |
| Fabio Stagnari, Michele Pisante | The critical period for weed competition in French bean (Phaseolus vulgarisL.) in Mediterranean areas | 2011 | Crop Protection | https://www.sciencedirect.com/science/article/pii/S026121941000325X |
| R. R. Weil, G.S. Belmont | Dry matter and nitrogen accumulation and partitioning in field grown winged bean | 1991 | Experimental Agriculture | https://www.cambridge.org/core/services/aop-cambridge-core/content/view/67126FEBBAC610E26EF462C6782BEFD1/S0014479700019049a.pdf/dry_matter_and_nitrogen_accumulation_and_partitioning_in_field_grown_winged_bean.pdf |
| Motior M. Rahman, Aminul M. Islam, Sofian M. Azirun, Amru N. Boyce | Tropical Legume Crop Rotation and Nitrogen Fertilizer Effects on Agronomic and Nitrogen Efficiency of Rice | 2014 | The Scientific World Journal | <https://www.hindawi.com/journals/tswj/2014/490841/> |
| T. Hymowitz, J. Boyd | Origin, Ethnobotany and Agricultural Potential of the Winged Bean: Psophocarpus tetragonolobus | 1977 | Economic Botany | https://www.jstor.org/stable/4253831?read-now=1&seq=1#page_scan_tab_contents |
| Fajri Anugroho, Makoto Kitou, Kazutoshi Kinjo, Norikazu Kobashigawa | Growth and Nutrient Accumulation of Winged Bean and Velvet Bean as Cover Crops in a Subtropical Region | 2010 | Plant Prod. Sci. | https://www.jstage.jst.go.jp/article/pps/13/4/13_4_360/_pdf |
| B. Ikhajiagbe, G. C. Mgbeze, H. A. Erhenhi | Growth and yield responses of Sphenostylis stenocarpa (Hochst ex A. Rich) Harms to phosphate enrichment of soil | 2009 | African Journal of Biotechnology | https://www.ajol.info/index.php/ajb/article/viewFile/59896/48170 |
| Adewale B. Daniel, Odoh N. Celestina | A Review on Genetic Resources, Diversity and Agronomy of African Yam Bean (Sphenostylis stenocarpa (Hochst. Ex A. Rich.) Harms): A Potential Future Food Crop | 2013 | Sustainable Agriculture Research | https://www.researchgate.net/publication/272801141_A_Review_on_Genetic_Resources_Diversity_and_Agronomy_of_African_Yam_Bean_Sphenostylis_stenocarpa_Hochst_Ex_A_Rich_Harms_A_Potential_Future_Food_Crop |
| Abigail Tettey | Nodulation, nitrogen fixation and diversity of the African yam bean rhizobia in four Ghanaian soils | 2014 | University of Ghana, College of Humanities, School of Arts, Department of Philosophy and Classics | http://ugspace.ug.edu.gh/handle/123456789/7097?show=full |
| E.O. Ohanmu, B. Ikhajiagbe, B.O. Edegbai | Nitrogen Distribution Pattern of African Yam Bean (Sphenostylis stenocarpa) exposed to Cadmium stress | 2018 | J. Appl. Sci. Environ. Manage. | <https://www.ajol.info/index.php/jasem> |
| G. C. Mgbeze, B. Ikhajiagbe | Growth and yield responses of Sphenostylis stenocarpa (Hochst ex. A Rich) Harms (African yam bean) to potassium application | 2010 | African Journal of Biotechnology | http://citeseerx.ist.psu.edu/viewdoc/download?doi=10.1.1.830.8415&rep=rep1&type=pdf |
| Beckley Ikhajiagbe, Joseph Kwesi Mensah | Genetic Assessment of Three Colour Variants of African Yam Bean[Sphenostylis Stenocarpa] Commonly Grown in the Midwestern Region of Nigeria | 2012 | International Journal of Modern Botany | http://article.sapub.org/pdf/10.5923.j.ijmb.20120202.01.pdf |
| C. G Okeke, S.I. Oluka, O. Oduma | Effect of Some Indigenous Legumes on Soil properties and Yield of Maize Crop in anultisol in South-Eastern Nigeria | 2016 | American Journal of Engineering Reasearch | https://www.researchgate.net/publication/299575336_Effect_of_Some_Indigenous_Legumes_on_Soil_properties_and_Yield_of_Maize_Crop_in_anultisol_in_South-Eastern_Nigeria |
| Daniel Kwasi Asare, Christian Kofi Anthonio, Lee Kheng Heng, Emmanuel Ofori Ayeh | Nodulation and Fixed Atmospheric Nitrogen of Some Local Lima Bean (Phaseolus lunatus L.) Cultivars Grown in a Coastal Savannah Environment | 2015 | Agricultural Sciences | https://file.scirp.org/Html/5-3001128_59597.htm |
| R. S. Mehta, B. S. Patel, S. S. Meena, R. S. Meena | Influence of nitrogen, phosphorus and bio-fertilizers on growth characters and yield of fenugreek (Trigonella foenum-graecum L.) | 2010 | Journal of Spices and Aromatic Crops | http://updatepublishing.com/journals/index.php/josac/article/view/607/569 |
| Neelesh Kapoor | Physiological, biochemical and molecular responses of fenugreek to salinity: elucidation of possible tolerance mechanism | 2016 | KUMAUN UNIVERSITY, NAINITAL, UTTARAKHAND, INDIA | http://ir.inflibnet.ac.in:8080/jspui/bitstream/10603/206426/9/neelesh%20ph.d.%20thesis%20_cd.pdf |
| Million Fikreselassie, Habtamu Zeleke, Nigussie Alemayehu | Genetic variability of Ethiopian fenugreek (Trigonella foenum-graecum L.) landrace | 2012 | Journal of Plant Breeding and Crop Science | https://academicjournals.org/journal/JPBCS/article-full-text-pdf/AA0E76810130 |
| K. M. Mccormick, Robert Michael Norton, H. A. Eagles | Phenotypic variation within a fenugreek (Trigonellafoenum-graecumL.) germplasm collection. II. Cultivar selection based on traits associated with seed yield | 2009 | Genetic Resources and Crop Evolution | https://www.researchgate.net/publication/225129845_Phenotypic_variation_within_a_fenugreek_Trigonella_foenum-graecum_L_germplasm_collection_II_Cultivar_selection_based_on_traits_associated_with_seed_yield |
| R.S. Mehta, B.S. Patel, S.S.Meena, G. Lal And R.Singh | Water dynamics and yield of fenugreek (Trigonella-foenium-graecum L.) as influenced by irrigation scheduling and weed management practices | 2014 | International J. Seed Spices | http://isss.ind.in/pdf/2014volume/7.pdf |
| Rajbir Singh, Dilip Kumar Kundu | Water use by horsegram (Macrotyloma uniflorum), greengram (Phaseolus radiatus) and sesame (Sesamum indicum) under shallow water-table condition in Orissa | 2003 | Indian Journal of Agricultural Sciences | https://www.researchgate.net/publication/289027790_Water_use_by_horsegram_Macrotyloma_uniflorum_greengram_Phaseolus_radiatus_and_sesame_Sesamum_indicum_under_shallow_water-table_condition_in_Orissa |
| H.C. Singhal, S.S. Tomar, B.R.Baraiya, R.S.Sikarwar, I.S. Tomar | Study of genetic divergence in horsegram (Macrotyloma uniflorum l.) | 2010 | Legume Res. | https://arccjournals.com/journal/legume-research-an-international-journal/ARCC1291 |
| N. H. Nam, y. S. Chauhan, c. Johansen | Comparison of extra-short-duration pigeonpea with short-season legumes under rainfed conditions on alfisols | 1993 | Experimental Agriculture | <https://www.cambridge.org/core/services/aop-cambridge-core/content/view/60764A64C495223A887B41AA737203B0/S0014479700020871a.pdf/comparison_of_extrashortduration_pigeonpea_with_shortseason_legumes_under_rainfed_conditions_on_alfisols.pdf> |
| Bolbhat Sadashiv N., Dhumal Kondiram N. | Effect of mutagens on dry biomass, root nodules, seed and biological yield and harvest index in M2 and M3 generation of horsegram (Macrotyloma uniflorum (Lam.) Verdc) | 2012 | International Journal of advanced scientific and technical research | http://www.rspublication.com/ijst/dec12/64.pdf |
| A. M. Abd El Moneim | Agronomic Potential of Three Vetches (Vicia spp.) Under Rainfed Conditions | 1993 | Journal of Agronomy and Crop Science | https://onlinelibrary.wiley.com/doi/epdf/10.1111/j.1439-037X.1993.tb01064.x |
| Alpaslan Kusvuran, Erhan Lami Parlak, Turan Sağlamtimur | Biomass Yield of Faba Bean (Vicia fabaL.) and its mixture with Some Grasses (Poaceae) | 2015 | Türk Tarim ve Doga Bilimleri | http://dergipark.gov.tr/download/issue-file/3058 |
| A.Fasheun, M.D.Dennett | Interception of radiation and growth efficiency in field beans (Vicia Faba L.) | 1982 | Agricultural Meteorology | https://www.sciencedirect.com/science/article/pii/0002157182900334 |
| Magdi T. Abdelhamid, Mahmoud M. B. Shokr, M. A. Bekheta | Growth, Root Characteristics, and Leaf Nutrients Accumulation of Four Faba Bean (Vicia faba L.) Cultivars Differing in Their Broomrape Tolerance and the Soil Properties in Relation to Salinity | 2010 | Communications in Soil Science and Plant Analysis | https://www.tandfonline.com/doi/abs/10.1080/00103624.2010.518263 |
| A. M. Abd El Moneim | Narbon Vetch (Vicia narbonensis L.): A Potential Feed Legume Crop for Dry Areas in West Asia | 1992 | Journal of Agronomy and Crop Science | https://onlinelibrary.wiley.com/doi/abs/10.1111/j.1439-037X.1992.tb01046.x |
| J.D. Berger, L.D. Robertson, P.S. Cocks | Agricultural potential of Mediterranean grain and forage legumes: Key differences between and within Vicia species in terms of phenology, yield,and agronomy give insight into plant adaptation to semi-arid environments | 2002 | Genetic Resources and Crop Evolution | https://www.researchgate.net/publication/225345418_Agricultural_potential_of_Mediterranean_grain_and_forage_legumes_Key_differences_between_and_within_Vicia_species_in_terms_of_phenology_yield_and_agronomy_give_insight_into_plant_adaptation_to_semi-ar |
| A. Larbia, A. M. Abd El-Moneim, H.Nakkoula, B.Jammal, S.Hassana | Intra-species variations in yield and quality determinants in Vicia species: 3. Common vetch (Vicia sativa ssp. sativa L.) | 2011 | Animal Feed Science and Technology | https://www.sciencedirect.com/science/article/pii/S0377840111000216 |
| J. P. Srivastava, C. P. Soni | Physiological Basis of Yield in Moth Bean [Vigna aconitifolia (Jacq.) Marechal] Under Rainfed Conditions | 1995 | Journal of Agronomy and Crop Science | https://onlinelibrary.wiley.com/doi/abs/10.1111/j.1439-037X.1995.tb00196.x |
| Uday Burman, B. K. Garcj, S. Kathju | Effect of spacing on seed yield and physiological traits in moth bean (Vigna aconitifolia) | 2002 | Indian Journal of Agricultural Sciences | http://krishikosh.egranth.ac.in/bitstream/1/2029046/1/ICAR-IJOAS-062.pdf#page=18 |
| J.C.Tarafdar, D.Kumar | Moth bean (Vigna aconitifolia) cultivars compared for root nutrient uptake efficiency in arid NW India | 2003 | Journal of Arid Environments | https://www.sciencedirect.com/science/article/pii/S0140196303000326 |
| K.G.Mohan Kumar | Seasonal influence on productivity potential in mothbean genotypes [Vigna aconitifolia (Jacq.) Marechal] | 2008 | UAS, Dharwad | http://krishikosh.egranth.ac.in/handle/1/82762 |
| J.C.Tarafdar, D.Kumar | Moth bean (Vigna aconitifolia) cultivars compared for root nutrient uptake efficiency in arid NW India | 2003 | Journal of Arid Environments | https://www.sciencedirect.com/science/article/pii/S0140196303000326 |
| Sonoko D. Kimura, Knut Schmidtke, Ryosuke Tajima, Koichi Yoshida, Hiroshi Nakashima, Rolf Rauber | Seasonal N uptake and N fixation by common and adzuki bean at various spacings | 2004 | Plant and Soil | https://www.researchgate.net/profile/Sonoko_Kimura/publication/248058740_Seasonal_N_uptake_and_N_2_fixation_by_common_and_adzuki_bean_at_various_spacings/links/5876001b08ae329d6224b91a/Seasonal-N-uptake-and-N-2-fixation-by-common-and-adzuki-bean-at-various-spacings.pdf |
| M. Vanaja, P. Ratnakumar, P. Vagheera, M. Jyothi, P. Raghuram Reddy, N. Jyothi Lakshmi, M. Maheshwari, S.K. Yadav | Initial growth responses of blackgram (Vigna mungo L. Hepper) under elevated CO2 and moisture stress | 2006 | PLANT SOIL ENVIRON | https://www.agriculturejournals.cz/publicFiles/50923.pdf |
| Baroowa B., Gogoi N. | Morpho-physiological and Yield responses of Black gram (Vignamungo L.) and Green gram (Vigna radiata L.) genotypes under Drought at different Growth stages | 2016 | Research Journal of Recent Sciences | https://www.researchgate.net/publication/294259182_Morpho-physiological_and_Yield_responses_of_Black_gram_Vignamungo_L_and_Green_gram_Vigna_radiata_L_genotypes_under_Drought_at_different_Growth_stages |
| A. Ghafoor, A. Sharif, Z. Ahmad, M. Azahid, M.A. Rabbani | Genetic diversity in blackgram (Vigna mungo L. Hepper) | 2001 | Field Crops Research | https://www.sciencedirect.com/science/article/pii/S0378429000001416 |
| J. A. Vera-Nunez, J. P. Infante-Santiago , V. Velasco Velasco , S. Salgado Garcia , D. J. Palma-Lopez , O. A. Grageda-Cabrera , R. Cardenas, J. J. Pena-Cabriales | Influence of P Fertilization on Biological Nitrogen Fixation in Herbaceous Legumes Grown in Acid Savannah Soils from the Tabasco State, Mexico | 2008 | Journal of Sustainable Agriculture | https://www.tandfonline.com/doi/abs/10.1300/J064v31n03_04 |
| E. J. Littleton, M. D. Dennett, J. Elston, J. L. Monteith | The growth and development of cowpeas (Vigna unguiculata) under tropical | 1979 | The Journal of Agricultural Science | https://www.cambridge.org/core/journals/journal-of-agricultural-science/article/growth-and-development-of-cowpeas-vigna-unguiculata-under-tropical-field-conditions-1-leaf-area/6BEF1E7C6B9D89F0B9874EAE1039ECEE |
| O. Babalola | Water relations of three cowpea cultivars (Vigna unguiculata, L.) | 1980 | Plant and Soil | https://link.springer.com/article/10.1007/BF02197953 |
| I.B.Y. Abdou Razakou, S. Addam Kiari, B. Mensah, R. Akromah | Water stress and water use efficiency in cowpea [Vigna unguiculata (L.) Walp.] under controlled environment | 2013 | International Journal of Agricultural Science Research | https://www.researchgate.net/publication/262724406_Water_stress_and_water_use_efficiency_in_cowpea_Vigna_unguiculata_L_Walp_under_controlled_environment |
| Ahmed M. El Naim And Abdelrhim A. Jabereldar | Effect of Plant density and Cultivar on Growth and Yield of Cowpea (Vigna unguiculata L.Walp) | 2010 | Australian Journal of Basic and Applied Sciences | https://www.researchgate.net/profile/Ahmed_El_Naim/publication/233945209_Effect_of_Plant_density_and_Cultivar_on_Growth_and_Yield_of_Cowpea_Vigna_unguiculata_LWalp/links/0fcfd50d336f5bb47c000000.pdf |
|  | Trends In Weed Management | 2018 | AGRILIT | https://www.agrilit.com/trends-weed-management/ |
| Aqsa Tabasum, Muhammad Saleem, Irum Aziz | Genetic variability, trait association and path analysis of yield and yield components in mungbean (Vigna radiata (l.) Wilczek) | 2010 | Pak. J. Bot. | http://www.pakbs.org/pjbot/PDFs/42(6)/PJB42(6)3915.pdf |
| S.S. Yadav, G. Bejiga | Lathyrus sativus L. | 2006 | PROTA | https://prota4u.org/database/protav8.asp?h=M36&t=Lathyrus,ochrus&p=Lathyrus+sativus# |
| G. Bejiga | Lens culinaris Medik. | 2006 | PROTA | https://prota4u.org/database/protav8.asp?h=M4&t=Lens,culinaris&p=Lens+culinaris#Synonyms |
| P.C.M. Jansen | Lupinus albus L. [Internet] Record from PROTA4U. | 2006 | PROTA | https://prota4u.org/database/protav8.asp?h=M4&t=Lupinus,albus&p=Lupinus+albus#Synonyms |
| L. López-Bellido & M. Fuentes | Lupinus L.. | 2006 | PROTA | <https://prota4u.org/prosea/view.aspx?id=3026> |
| M. Brink | Macrotyloma uniflorum (Lam.) Verdc. | 2006 | PROTA | https://prota4u.org/database/protav8.asp?h=M1,M17,M25,M26,M27,M28,M34,M36,M4,M7&t=Macrotyloma&p=Macrotyloma+uniflorum#Protologue |
| N. Wulijarni-Soetjipto & R.F. Maligalig | Mucuna pruriens (L.) DC. cv. group Utilis. | 1997 | PROTSEA | https://prota4u.org/prosea/view.aspx?id=63 |
| P.C.M. Jansen | Phaseolus acutifolius A. Gray. | 1989 | PROTSEA | https://prota4u.org/prosea/view.aspx?id=3311 |
| K.K. Mogotsi | Phaseolus acutifolius A.Gray. [Internet] Record from PROTA4U. | 2006 | PROTA | https://prota4u.org/database/protav8.asp?h=M4&t=Phaseolus&p=Phaseolus+acutifolius#Synonyms |
| M. Brink | Phaseolus coccineus L. [Internet] Record from PROTA4U. | 2006 | PROTA | https://prota4u.org/database/protav8.asp?h=M4&t=Phaseolus&p=Phaseolus+coccineus#Synonyms |
| J.P. Baudoin | Phaseolus lunatus L. [Internet] Record from PROTA4U. | 2006 | PROTA | <https://prota4u.org/database/protav8.asp?h=M4&t=Phaseolus&p=Phaseolus+lunatus#Synonyms> |
| J.P. Baudoin | Phaseolus lunatus L. [Internet] Record from PROTA4U. | 1989 | PROTSEA | https://prota4u.org/prosea/view.aspx?id=3313 |
| C.-M. Messiaen, A.A. Seif | Phaseolus vulgaris L. (French bean) [Internet] Record from PROTA4U. | 2004 | PROTA | <https://prota4u.org/database/protav8.asp?h=M4&t=Phaseolus&p=Phaseolus+vulgaris+(French+bean)#Synonyms> |
| C.-M. Messiaen, A.A. Seif, M. Jarso, G. Keneni | Pisum sativum L. [Internet] Record from PROTA4U. tropicale), Wageningen, Netherlands. | 2006 | PROTA | https://prota4u.org/database/protav8.asp?h=M4&t=Pisum&p=Pisum+sativum#Synonyms |
| G.J.H. Grubben | Psophocarpus tetragonolobus (L.) DC. [Internet] Record from PROTA4U. | 2004 | PROTA | https://prota4u.org/database/protav8.asp?h=M4&t=Psophocarpus,tetragonolobus&p=Psophocarpus+tetragonolobus#Synonyms |
| Ezueh, M.I. | African yam bean as a crop in Nigeria. | 1984 | World Crops |  |
| Popoola, J. O., A. E. Adegbite, O. O. Obembe, B. D. Adewale, B. O. Odu | Morphological intraspecific variabilities in African yam bean (AYB) (Sphenostylis stenocarpa Ex. A. Rich) Harms | 2011 | Scientific Research and Essay | https://www.researchgate.net/publication/260301001_Morphological_intraspecific_variabilities_in_African_yam_bean_AYB_Sphenostylis_stenocarpa_Ex_A_Rich_Harms |
| Ken Fern | Useful Tropical Plants Database. |  |  | Tropical.Theferns.Info |
| Adewale B. Daniel, Odoh N. Celestina | A Review on Genetic Resources, Diversity and Agronomy of African Yam Bean (Sphenostylis stenocarpa(Hochst. Ex A. Rich.) Harms): A Potential Future Food Crop | 2013 | Sustainable Agriculture Research | https://www.researchgate.net/publication/272801141_A_Review_on_Genetic_Resources_Diversity_and_Agronomy_of_African_Yam_Bean_Sphenostylis_stenocarpa_Hochst_Ex_A_Rich_Harms_A_Potential_Future_Food_Crop |
| C.S. Tawan & N. Wulijarni-Soetjipto | Trigonella foenum-graecum L.. | 1999 | PROTSEA | https://prota4u.org/prosea/view.aspx?id=601 |
| D. Enneking, A. Lahlou, A. Noutfia, M. Bounejmate | A note on Vicia ervilia cultivation utilisation and toxicity in Morocco | 1995 | Al Awamia | http://webagris.inra.org.ma/doc/awamia/08911.pdf |
| Salama El Fatehi, Gilles Béna, Abdelkarim Filali-Maltouf, Mohammed Ater | Variation in yield component, phenology and morphological traits among Moroccan bitter vetch landraces Vicia ervilia(L.) Wild | 2014 | African Journal of Agricultural Research | https://academicjournals.org/article/article1402415986_El%20Fatehi%20et%20al.pdf |
| Dzyubenko N. I., Dzyubenko E. A. | Interactive Agricultural Ecological Atlas of Russia and Neighboring Countries : Vicia ervilia L. - Bitter vetch. | 2003-2009 | Interactiv Agricultural Ecological Atlas of Russia and Neighboring Countries | http://www.agroatlas.ru/en/content/related/Vicia_ervilia/index.html |
| N.F.Miller, D Enneking | BitterVetch(Vicia ervilia)Ancient Medicinal Crop and Farmers' Favorite for Feeding Livestock | 2014 | in : New lives dor ancient and extinct Crops. Ed. P.E. Minnis | https://www.researchgate.net/publication/262048759_Bitter_vetch_Vicia_ervilia_-_ancient_medicinal_crop_and_farmers_favorite_for_feeding_livestock |
| Wikipedia | Vicia ervilia |  |  | https://fr.wikipedia.org/wiki/Wikip%C3%A9dia:Accueil_principal |
| Papastylianou, I. | Amount of nitrogen fixed by forage, pasture and grain legumes in Cyprus, estimated by the A-value and a modified difference method. | 1987 | Plant and Soil | https://link.springer.com/article/10.1007/BF02370620 |
| Neyestani, E. | Assessment Of Grain Yield And Plant Characteristics In Vetch (Vicia Ervilia L.) Genotypes In Cold Dryland Conditions Of Northern Khorasan. | 2009 | Iranian Journal of Crop Sciences | http://agrobreedjournal.ir/browse.php?a_id=200&sid=1&slc_lang=en |
| I. Papastylianou | The Role of Legumes in Agricultural Production in Cyprus | 1988 | Springer | https://link.springer.com/chapter/10.1007/978-94-009-1387-5_7 |
| M. Jarso, G. Keneni | Vicia faba L. [Internet] Record from PROTA4U. | 2006 | PROTA | https://prota4u.org/database/protav8.asp?h=M4&t=Vicia&p=Vicia+faba#Synonyms |
| Heuzé V., Tran G., Lebas F. | Narbon vetch (Vicia narbonensis). | 2015 | Feedipedia | <http://www.efloras.org/florataxon.aspx?flora_id=5&taxon_id=250065305> |
| A. A. Al-Doss, A. M. Assaeed, A. S. Soliman | Growth Characters and Yield of Some Selected Lines of Common and Narbon Vetch | 1996 | Agr. Res. Center | https://www.feedipedia.org/node/240 |
| Heuzé V., Tran G., Baumont R | *Common vetch (Vicia sativa)*. | 2015 | Feedipedia | https://pdfs.semanticscholar.org/c2f1/d120e082f52412c5f33f729931e2a651af5c.pdf |
| M. Brink, P.C.M. Jansen | Vigna aconitifolia (Jacq.) Maréchal. [Internet] Record from PROTA4U. | 2006 | PROTA | https://www.feedipedia.org/node/239 |
| P.C.M. Jansen | Vigna angularis (Willd.) Ohwi & H.Ohashi. [Internet] Record from PROTA4U. | 2006 | PROTA | https://prota4u.org/database/protav8.asp?h=M4&t=Vigna&p=Vigna+aconitifolia#Synonyms |
| Akito Kaga, Takehisa Isemura, Norihiko Tomooka, Duncan A. Vaughan | The Genetics of Domestication of the Azuki Bean (Vigna angularis) | 2008 | Genetics | https://prota4u.org/database/protav8.asp?h=M4&t=Vigna&p=Vigna+angularis#Synonyms |
| P.C.M. Jansen | Vigna mungo (L.) Hepper. [Internet] Record from PROTA4U. | 2006 | PROTA | https://www.ncbi.nlm.nih.gov/pmc/articles/PMC2248364/ |
| R. Rajerison | Vigna umbellata (Thunb.) Ohwi & H.Ohashi. [Internet] Record from PROTA4U. | 2006 | PROTA | https://prota4u.org/database/protav8.asp?h=M4&t=Vigna&p=Vigna+mungo#Synonyms |
| R. Madamba, G.J.H. Grubben, I.K. Asante, R. Akromah | Vigna unguiculata (L.) Walp. [Internet] Record from PROTA4U. | 2006 | PROTA | https://prota4u.org/database/protav8.asp?h=M4&t=Vigna&p=Vigna+umbellata#Synonyms |
| K.K. Mogotsi | Vigna radiata (L.) R.Wilczek. [Internet] Record from PROTA4U. | 2006 | PROTA | <https://prota4u.org/database/protav8.asp?h=M4&t=Vigna&p=Vigna+unguiculata#Synonyms> |
| M.J. Bell, R. Shorter, R. Mayer | Cultivar and environmental effects on growth and development of peanuts (Arachis hypogaea L. ). I. Emergence and flowering | 1991 | Field Crops Research | <https://prota4u.org/database/protav8.asp?h=M4&t=Vigna&p=Vigna+radiata#Synonyms> |
| Salmina N. Mokgehle, Felix D. Dakora, Cherian Mathews | Variation in N2 fixation and N contribution by 25 groundnut (Arachis hypogaea L.) varieties grown in different agro-ecologies, measured using 15N natural abundance | 2014 | Agriculture, Ecosystems and Environment | https://ac.els-cdn.com/037842909190019R/1-s2.0-037842909190019R-main.pdf?_tid=e66a27be-4ba1-40c0-bfaa-9af9f4e6e399&acdnat=1547558332_d74ca3d2ea2a41beeded498d0c3883a6 |
| R. C. Nageswara Rao, H. S. Talwar And G. C. Wright | Rapid Assessment of SpeciÆc Leaf Area and Leaf Nitrogen in Peanut(Arachis hypogaeaL.) using a Chlorophyll Meter | 2001 | J. Agronomy & Crop Science | https://www.sciencedirect.com/science/article/pii/S0167880914003041 |
| P. Songsri, S. Jogloy, C.C. Holbrook,, T. Kesmala, N. Vorasoot, C. Akkasaeng, A. Patanothai | Association of root, specific leaf area and SPAD chlorophyll meter reading to water use efficiency of peanut under different available soil water | 2009 | Agricultural Water Management | https://s3.amazonaws.com/academia.edu.documents/41359772/Rapid_Assessment_of_Specific_Leaf_Area_a20160120-20049-1hcs85o.pdf?AWSAccessKeyId=AKIAIWOWYYGZ2Y53UL3A&Expires=1547564099&Signature=FuN%2F6u%2FpufyTaPnDFMUUtM46%2BD8%3D&response-content-disposition=inline%3B%20filename%3DRapid_Assessment_of_Specific_Leaf_Area_a.pdf |
| K. F. El-Sahhar, Kh. S. Emara, W.A Ali | Comparative Systematic Studies of Astragalus L. In Flora of Arab Republic of Egypt and Syrian Arab Republic: Seed Features and Germination | 2013 | Research Journal of Agriculture and Biological Sciences | https://pubag.nal.usda.gov/pubag/downloadPDF.xhtml?id=28021&content=PDF |
| Andrés Ivorra | Astragalus edulis Bunge |  | almerinatura | <http://www.aensiweb.net/AENSIWEB/rjabs/rjabs/2013/79-88.pdf> |
| Craine, J. M., A. J. Elmore, M. P. M. Aidar, M. Bustamante, T. E. Dawson, E. A. Hobbie, A. Kahmen, M. C. Mack, K. K. Mclauchlan, A. Michelsen, G. B. Nardoto, L. H. Pardo, J. Penuelas, P. B. Reich, E. A. G. Schuur, W. D. Stock, P. H. Templer, R. A. Virginia, J. M. Welker, And I. J. Wright. | Global patterns of foliar nitrogen isotopes and their relationships with climate, mycorrhizal fungi, foliar nutrient concentrations, and nitrogen availability. | 2009 | New phytologist | http://www.almerinatura.com/joyas/astragalus-edulis.html |
| Kerkhoff, A. J., W. F. Fagan, J. J. Elser, And B. J. Enquist. | Phylogenetic and growth form variation in the scaling of nitrogen and phosphorus in the seed plants. | 2006 | American Naturalist | https://www.journals.uchicago.edu/doi/10.1086/507879 |
| Wright, I. J., P. B. Reich, M. Westoby, D. D. Ackerly, Z. Baruch, F. Bongers, J. Cavender-Bares, T. Chapin, J. H. C. Cornelissen, M. Diemer, J. Flexas, E. Garnier, P. K. Groom, J. Gulias, K. Hikosaka, B. B. Lamont, T. Lee, W. Lee, C. Lusk, J. J. Midgley, M. L. Navas, U. Niinemets, J. Oleksyn, N. Osada, H. Poorter, P. Poot, L. Prior, V. I. Pyankov, C. Roumet, S. C. Thomas, M. G. Tjoelker, E. J. Veneklaas, And R. Villar. | The worldwide leaf economics spectrum. | 2004 | Nature | https://www.nature.com/articles/nature02403 |
| Kattge, J., W. Knorr, T. Raddatz, And C. Wirth. | Quantifying photosynthetic capacity and its relationship to leaf nitrogen content for global-scale terrestrial biosphere models. | 2009 | Global Change Biology | https://onlinelibrary.wiley.com/doi/10.1111/j.1365-2486.2008.01744.x |
| Wenxuan Han, Yahan Chen, Fang-Jie Zhao, Luying Tang, Rongfeng Jiang And Fusuo Zhang, | Floral, climatic and soil pH controls on leaf ash content in China’s terrestrial plants. | 2012 | Global Ecology and Biogeography | https://onlinelibrary.wiley.com/doi/abs/10.1111/j.1466-8238.2011.00677.x |
| Prentice, I.C., Meng, T., Wang, H., Harrison, S.P., Ni, J., Wang, G. | Evidence for a universal scaling relationship of leaf CO2 drawdown along a moisture gradient. | 2011 | New phytologist | https://nph.onlinelibrary.wiley.com/doi/10.1111/j.1469-8137.2010.03579.x |
| Tribouillois H, Fort F, Cruz P, Charles R, Flores O, Garnier E, Et Al. | A Functional Characterisation of a Wide Range of Cover Crop Species: Growth and Nitrogen Acquisition Rates, Leaf Traits and Ecological Strategies. | 2015 | PLoS ONE | https://journals.plos.org/plosone/article?id=10.1371/journal.pone.0122156 |
| Maire V, Ian J. Wright, I. Colin Prentice, Niels H. Batjes, Radika Bhaskar, Peter M. Van Bodegom, Will K. Cornwell, David Ellsworth, Ülo Niinemets, Alejandro Ordoñez, Peter B. Reich, Louis S. Santiago | Global effect of soil and climate on leaf photosynthetic traits and rates. | 2015 | Global Ecology and Biogeography | https://onlinelibrary.wiley.com/doi/abs/10.1111/geb.12296 |
| Walker, A.P | A Global Data Set of Leaf Photosynthetic Rates, Leaf N and P, and Specific Leaf Area | 2014 | Data set. Available on-line | https://daac.ornl.gov/cgi-bin/dsviewer.pl?ds_id=1224 |
| Royal Botanical Gardens Kew. | Seed Information Database (SID). | 2008 | Data set. Available on-line | https://data.kew.org/sid/ |
| Kleyer, M., R. M. Bekker, I. C. Knevel, J. P. Bakker, K. Thompson, M. Sonnenschein, P. Poschlod, J. M. Van Groenendael, L. Klimes, J. Klimesova, S. Klotz, G. M. Rusch, Hermy, M. , D. Adriaens, G. Boedeltje, B. Bossuyt, A. Dannemann, P. Endels, L. Götzenberger, J. G. Hodgson, A.-K. Jackel, I. Kühn, D. Kunzmann, W. A. Ozinga, C. Römermann, M. Stadler, J. Schlegelmilch, H. J. Steendam, O. Tackenberg, B. Wilmann, J. H. C. Cornelissen, O. Eriksson, E. Garnier, And B. Peco. | The LEDA Traitbase: a database of life-history traits of the Northwest European flora | 2008 | Journal of Ecology | https://besjournals.onlinelibrary.wiley.com/doi/10.1111/j.1365-2745.2008.01430.x |
| Vile, D. | Significations fonctionnelle et ecologique des traits des especes vegetales: exemple dans une succession post-cultural mediterraneenne et generalisations, PHD Thesis. | 2005 | PHD thesis Montpellier University | https://www.theses.fr/2005MON20109 |
| Ruben Velez-Colon, Steplien A. Garrison | Growth, maturity and flowering of pigeon peas, Cajanus cajan Millsp., at high latitudes | 1989 | J. Agric. Univ. P.R. | https://revistas.upr.edu/index.php/jaupr/article/view/6460 |
| Guriqbal Singh, H.S. Sekhon | Integrated Weed Management in Pigeonpea [Cajanus cajan (L.) Millsp.] | 2013 | World Journal of Agricultural Sciences | https://revistas.upr.edu/index.php/jaupr/article/viewFile/6460/5114 |
| C.D.J. Kessler | An agronomic evaluation of jackbean {canavalia ensiformis) in yucatan, mexico. I. Plant density | 1990 | Experimental Agriculture | https://pdfs.semanticscholar.org/eddb/2408bb19c68cfad1e65151698a709cab0431.pdf |
| Margarita G. Ramos, Maria Antonieta A. Villatoro, Segundo Urquiaga, Bruno J.R. Alves, Robert M. Boddey | Quantification of the contribution of biological nitrogen fixation to tropical green manure crops and the residual benefit to a subsequent maize crop using 15 N-isotope techniques | 2001 | Journal of Biotechnology | https://www.cambridge.org/core/services/aop-cambridge-core/content/view/E876319277F8D1A4CC2289D569C97887/S0014479700015350a.pdf/an_agronomic_evaluation_of_jackbean_canavalia_ensiformis_in_yucatan_mexico_i_plant_density.pdf |
| C.Swortmann, B.Dmcintyre, C.Kkaizzic | Annual soil improving legumes: agronomic effectiveness, nutrient uptake, nitrogen fixation and water use | 2000 | Field crop research | https://ac.els-cdn.com/S0168165601003352/1-s2.0-S0168165601003352-main.pdf?_tid=da8364cf-b759-41d3-a100-a4f1a44a6583&acdnat=1547626545_85f7895b23fa3b2ac57ea281e24803da |
| Ekanayake, S., Jansz, E. R., & Nair, B. M. | Literature review of an underutilized legume: Canavalia gladiataL. | 2000 | Plant Foods for Human Nutrition | https://www.sciencedirect.com/science/article/pii/S0378429000001131 |
| C.H. Bosch | anavalia gladiata (Jacq.) DC. | 2006 | PROTA | <https://link.springer.com/article/10.1023%2FA%3A1008119107738> |
| J. Evans | Symbiosis, nitrogen and dry matter distributionin chickpea (cicer arietinum) | 1982 | Experimental Agriculture | <https://prota4u.org/database/protav8.asp?h=M4&t=Vigna&p=Vigna+radiata#Synonyms> |
| Navkiran Randhawa, Jagmeet Kaur, Sarvjeet Singh And Inderjit Singh | Growth and yield in chickpea (Cicer arietinumL.) genotypes in response to water stress | 2014 | African Journal of Agricultural Research | <https://www.cambridge.org/core/services/aop-cambridge-core/content/view/35123568D293A48AF800BB47EF4CA9A6/S0014479700000764a.pdf/symbiosis_nitrogen_and_dry_matter_distribution_in_chickpea_cicer_arietinum.pdf> |
| Shubhra Barwa,Balaram Pani, Leena Shakya | Influence of Phosphorus on Dry matter Partitioning and Nutrient Allocation in Clusterbean under Water deficit | 2017 | IJSAR | https://www.researchgate.net/publication/261181193_Growth_and_yield_of_chickpeaCicer_arietinum_L_genotypes_in_response_to_water_stress |
| Muhammad Aamir Iqbal | Cluster Bean (Cyamopsis tetragonoloba L.) Germination and Seedling Growth as Influenced by Seed Invigoration Techniques | 2015 | American-Eurasian J. Agric.&Environ. Sci. | http://www.ijsar.in/Admin/pdf/463.pdf |
| Heuzé V., Tran G., Sauvant D., Renaudeau D. , Bastianelli D. , Lebas F. | Lablab (Lablab purpureus). | 2016 | Feedipedia | https://www.researchgate.net/publication/278020014_Cluster_Bean_Cyamopsis_tetragonoloba_L_Germination_and_Seedling_Growth_as_Influenced_by_Seed_Invigoration_Techniques |
| T. A. Amole, B. O. Oduguwa, O. Shittu, A. Famakinde, N. Okwelum, V. O. A. Ojo, P. A. Dele, O. J. Idowu, B. Ogunlolu, A. O. Adebiyi | Herbage yield and quality of Labab purpureus during the late dry season in western Nigeria | 2013 | Slovak J; Anim. Sci | http://www.feedipedia.org/node/297 |
| R.C.Muchow | Phenology, seed yield and water use of grain legumes grown under different soil water regimes in a semi-arid tropical environment | 1985 | field Crops Research | http://www.cvzv.sk/slju/13_1/Amole.pdf |
| Castroviejo S Real Jardín Botánico (Spain | Flora ibérica : plantas vasculares de la Península Ibérica e Islas Baleares | 1986 | Real Jardín Botánico, C.S.I.C | https://ac.els-cdn.com/0378429085900930/1-s2.0-0378429085900930-main.pdf?_tid=8ba39f6d-f1c2-49db-9a3b-901d07355872&acdnat=1547642397_2143e2ee3ee0a368a4526a8b881d92e9 |
| K. H. M. Siddique, S. P. Loss, K. L. Regan, R. L. Jettner | Adaptation and seed yield of cool season grain legumes inMediterranean environments of south-western Australia | 1999 | Aust. J. Agric. Res. | <http://www.floraiberica.es/floraiberica/texto/pdfs/07_35%20Lathyrus.pdf> |
| P.G. Americanos And D.N. Droushiotis | Herbicides for forage legumes grown forhay under rainfed conditions in a mediterranean environment | 1999 | AGRICULTURAL RESEARCH INSTITUTE MINISTRY OF AGRICULTURE, NATURAL RESOURCES AND THE ENVIRONMENT | https://www.researchgate.net/publication/234520392_Adaptation_and_seed_yield_of_cool_season_grain_legumes_in_Mediterranean_environments_of_south-western_Australia |
| Felix May | Spatial models of plant diversity and plant functional traits – towards a better understanding of plant community dynamics in fragmented landscapes | 2013 | Plant Ecology & Nature Conservation University Potsdam |  |
| Valentine A Aletor, Ali Abd El-Moneim, Anthony V. Goodchild | Evaluation of the Seeds of Selected Lines of Three Lathyrus spp for p-N-Oxalylamino-L- Alanine (BOAA), Tannins, Trypsin Inhibitor Activity and Certain In-vitro Characteristics | 1994 | J. Sci. Food Agric. | https://publishup.uni-potsdam.de/opus4-ubp/frontdoor/deliver/index/docId/6648/file/may_felix_2013_10_30.pdf |
| M Hadjipanayiotou, S Economides | Chemical composition, in situ degradability and amino acid composition of protein supplements fed to livestock and poultry in Cyprus | 2001 | Livestock research for Rural Developement | <https://onlinelibrary.wiley.com/doi/pdf/10.1002/jsfa.2740650204> |
| Büchi L Gebhard C Liebisch F Sinaj S Ramseier H Et. Al | Accumulation of biologically fixed nitrogen by legumes cultivated as cover crops in Switzerland | 2015 | Plant and Soil | https://www.lrrd.cipav.org.co/lrrd13/6/hadj136.htm |
| D. A. Wall, G. H. Friesen And D. Dryden | Effect of herbicides and annual weeds on the yield and seed quality of lathyrus (Lathyrus sativus L.) | 1988 | Crop Protection | http://ecocrop.fao.org/ecocrop/srv/en/cropView?id=7164 |
| Alireza Pirzad And Sevil Mohammadzade | The effects of drought stress and zeolites on morpho - physiological traits of Lathyrus sativus l. (grass pea) | 2018 | plant cell biotechnology and molecular biology | https://www.researchgate.net/publication/275580204_Accumulation_of_biologically_fixed_nitrogen_by_legumes_cultivated_as_cover_crops_in_Switzerland |
| R.J. Haynes , R.J. Martin, K.M. Goh | Nitrogen fixation, accumulation of soil nitrogen and nitrogen balance for some field-grown legume crops | 1993 | Field Crops Research | https://ac.els-cdn.com/0261219488900051/1-s2.0-0261219488900051-main.pdf?_tid=bee91e7f-c2a0-4bb5-8647-f7736248a192&acdnat=1547653032_a3c09967a23f18f673fc1391bb602d35 |
| R. Mcvicar, P. Mccall, C. Brenzil, S. Hartley, K. Panchuk, P. Mooleki, A. Vandenberg, S. Banniza | Lentils in Saskatchewan. Fact Sheet. | 2017 | government of saskatchewan |  |
| Saeed Reza Hosseinzadeh & Raheleh Ahmadpour | Evaluation of vermicompost fertilizer application on growth, nutrient uptake and photosynthetic pigments of lentil (Lens culinaris Medik.) under moisture deficiency conditions | 2018 | Journal of Plant Nutrition | https://ac.els-cdn.com/0378429093901419/1-s2.0-0378429093901419-main.pdf?_tid=b60bb43c-f581-48f5-b874-01791c34b6e9&acdnat=1547732718_5d5ec42c4a18fe7e79fa2de3033e073e |
| S.A. Ansari, Samiullah, M.M.R.K. Afridi And Nafees A. Khan | Response of Field-Grown Lentil to Pre-Sowing Seed Enrichment with Pyridoxine | 1990 | Field Crops Research | <http://publications.gov.sk.ca/documents/20/86381-LentilsinSaskatchewan.pdf> |
| Jamin A. Smitchger, Ian C. Burke, And Joseph P. Yenish | The Critical Period of Weed Control in Lentil (Lens culinaris) in thePacific Northwest | 2012 | Weed Science | <https://doi.org/10.1080/01904167.2018.1450419> |
| GRDC-Grain Research & Developpment Coprporation | Growth note- Lentil | 2017 |  | <https://ac.els-cdn.com/037842909090096T/1-s2.0-037842909090096T-main.pdf?_tid=e60f4eef-254c-4ccc-85ea-a481e7c187fb&acdnat=1547804616_4ea7ff9bbd0834db8b48a1182cac4a1e> |
| Teklay Abebe Teferi, Muruts Legesse And Tsehaye Birhane | Searching and Testing of White Lupine (Lupinus albus L.) for Adaptation And Resistant to Crenate Broomrape in Tigray, Ethiopia | 2015 | World Journal of Agricultural Sciences | <https://doi.org/10.1614/WS-D-11-00069.1> |
| J. M. Pozuelo, M. Fernandez‐Pascual, M. M. Lucas, M. R. De Felipe | Effect of eight herbicides from five different chemical groups on nitrogen fixation and grain yield in Lupinus albus L. grown in semi‐arid zones | 1989 | Weed Research | <https://grdc.com.au/__data/assets/pdf_file/0020/243281/GRDC-GrowNotes-Lentil-Southern.pdf> |
| Catherine D. Campbell* & Rowan F. Sage | Interactions between the effects of atmospheric CO2 content and P nutrition on photosynthesis in white lupin (Lupinus albus L.) | 2006 | Plant, Cell and Environment | https://idosi.org/wjas/wjas11(6)15/3.pdf |
| R.E. Zanvettor, A.C. Ravelo And A. M. Planchuelo | Leaf area, dry matter and grain yield of white lupins under rainfed and irrigated conditions |  | Facultad de C. Agropecuarias, Univ. Nac. de Córdoba C.C. 509 Córdoba (5000) Argentina |  |
| M Dracup, C Davies And H Tapscott | Temperature and water requirements for germination and emergence of lupin | 1993 | Australian Journal of Experimental Agriculture | <https://onlinelibrary.wiley.com/doi/pdf/10.1111/j.1365-3040.2005.01464.x> |
| D. Lemerle, B. Verbeek, N. Coombes | Losses in grain yield of winter crops from Lolium rigidum competition depend on crop species, cultivar and season | 1995 | Weed Research | <http://www.sbagro.org.br/bibliotecavirtual/arquivos/617.pdf> |
| M.L. Rodrigues, C.M.A. Pacheco And M.M. Chaves | Soil-plant water relations, root distribution and biomass partitioning in Lupinus albus L. under drought condition | 1995 | Journal of Experimental Botany | https://www.publish.csiro.au/an/EA9930759 |
| Rehana Asghar, Darleen A. Demason | Developmental changes in the cotyledons of Lupinus luteus l. During and after germination | 1990 | American Journal of Botany | <https://onlinelibrary.wiley.com/doi/abs/10.1111/j.1365-3180.1995.tb01648.x> |
| C. Tang, M. J. Unkovich And J. W. Bowden | Factors affecting soil acidification under legumes. III. Acid production by N2-fixing legumes as influenced by nitrate supply | 1999 | New phytologist | https://watermark.silverchair.com/46-8-947.pdf |
| Ruth J. Eastwood, Colin E. Hughes | 878. Lupinus mutabilis | 2018 | Curtis's Botanical Magazine | <https://bsapubs.onlinelibrary.wiley.com/doi/pdf/10.1002/j.1537-2197.1990.tb11386.x> |
| Suzanne Ashworth, Kent Whealy | Seed to Seed: Seed Saving and Growing Techniques for the Vegetable Gardener | 2002 |  | <https://nph.onlinelibrary.wiley.com/doi/pdf/10.1046/j.1469-8137.1999.00475.x> |
| J.R. Teasdale,L.O. Brandsæter,A. Calegari And F. Skora Neto | Chapter 4 : Cover Crops and Weed Management | 2007 | in : Non-chemical Weed Management Principles, Concepts and Technology. Ed. Mahesh K. Upadhyaya and Robert E. Blackshaw | <https://onlinelibrary.wiley.com/doi/pdf/10.1111/curt.12233> |
| P.Gurumoorthi, S.Senthil Kumar, V.Vadivel, And K.Janardhanan | Studies on agrobotanical charecters of different accessions of velvet bean collected from western ghats, south india | 2003 | Tropical and Subtropical Agroecosystems | https://books.google.fr/books?id=N08guuBXT5gC&pg=PA143&lpg=PA143&dq=lupinus+mutabilis+days+to+emergence&source=bl&ots=ZQwG_eorqf&sig=ACfU3U3CsOI7S_60B2H8yXc9Koj3b4UZUg&hl=fr&sa=X&ved=2ahUKEwin_qDD1PffAhVJzRoKHTB6D3AQ6AEwC3oECAAQAQ#v=onepage&q=lupinus%20mutabilis%20days%20to%20emergence&f=false |
| Jose Polania, Charlotte Poschenrieder, Idupulapati Rao, And Stephen Beebe | Estimation of phenotypic variability in symbiotic nitrogen fixation ability of common bean under drought stress using 15N natural abundance in grain | 2016 | European Journal of Agronomy | <https://www.researchgate.net/profile/Bruce_Maxwell/publication/237504599_2_Understanding_Weed-Crop_Interactions_to_Manage_Weed_Problems/links/0a85e5342b55c59785000000/2-Understanding-Weed-Crop-Interactions-to-Manage-Weed-Problems.pdf#page=59> |
| Chris A. Shisanya And Nkanata M. Gitonga | Evaluation of nitrogen fixation using15N dilution methods and economy of a maize-tepary bean intercrop farming system in semi-arid SE-Kenya | 2007 | Advances in integrated Soil Fertility Managment in Sub-saharan Africa | http://citeseerx.ist.psu.edu/viewdoc/download?doi=10.1.1.596.8868&rep=rep1&type=pdf |
| Agnieszka Hanaka, Waldemar Maksymiec, Wiesław Bednarek | The effect of methyl jasmonate on selected physiological parameters of copper-treated Phaseolus coccineus plants | 2015 | Plant Growth Regul | https://www.ncbi.nlm.nih.gov/pmc/articles/PMC4998141/ |
| NZ Jovanovic And JG Annandale | Crop growth model parameters of 19 summer vegetable cultivars for use in mechanistic irrigation scheduling models | 2000 | Water S.A | http://14.139.186.108/jspui/bitstream/123456789/242/37/36.pdf |
| Shruthi, M K | Effect Of Foliar Application Of Water Soluble Npk Fertilizer On Growth, Yield And Quality Of Lima Bean (Phaseolus lunatus L.) | 2013 | University of Agricultural Sciences, GKVK | <https://link.springer.com/article/10.1007/s10725-015-0048-8> |
| L. H. Ziska, A. E. Hall, R. M. Hoover | Irrigation management methods for reducing water use of cowpea (Vigna unguiculata [L.] Walp.) and lima bean (Phaseolus lunatus L.) while maintaining seed yield at maximum levels | 1985 | Irrig Sci | http://www.wrc.org.za |
| Robert E. Blackshaw, Louis J. Molnar, H.-Henning Muendel, Gilles Saindon, And Xiangju Li | Integration of Cropping Practices and Herbicides Improves Weed Management in Dry Bean (Phaseolus vulgaris) | 2000 | Weed Technology | http://krishikosh.egranth.ac.in/handle/1/84425 |
| Chris M. Boerboom And Frank L. Young | Effect of Postplant Tillage and Crop Density on Broadleaf Weed Control in Dry Pea (Pisum sativum) and Lentil (Lens culinaris) | 1995 | Weed Technology | <https://link.springer.com/article/10.1007/BF00262468> |
| Wai Wai Lwin And Thanda Aye | Analyzed Growth of Psophocarpus Tetragonolobus (L.) DC. Treated by Fertilizer, Pruning and Photoperiod | 2016 | University of Y | https://bioone.org/journals/weed-technology/volume-14/issue-2/0890-037X(2000)014%5b0327%3aIOCPAH%5d2.0.CO%3b2/Integration-of-Cropping-Practices-and-Herbicides-Improves-Weed-Management-in/10.1614/0890-037X(2000)014[0327:IOCPAH]2.0.CO;2.short |
| W. Erskine | Heritability and combining ability of vegetative and phenological characters of winged beans (Psophocarpus tetragonolobus (L.) D.C.) | 1981 | J. agric. Sci. | https://www.cambridge.org/core/journals/weed-technology/article/effect-of-postplant-tillage-and-crop-density-on-broadleaf-weed-control-in-dry-pea-pisum-sativum-and-lentil-lens-culinaris/4BE304DB07A3C85C93A7C2EC7BCACEB6 |
| E.E. Iruthayathas And H.M.W. Herath | Nodule formation and distribution during the establish- ment stageof six selections of winged bean | 1981 | scientia horticulturae | https://uyr.uy.edu.mm/bitstream/handle/123456789/599/Analyzed%20Growth%20of%20Psophocarpus%20Tetragonolobus%20(L.)%20DC.%20Treated%20by%20Fertilizer,....pdf?sequence=1 |
| Q.N. Wong, F. Massawe And S. Mayes | Improving winged bean (Psophocarpus tetragonolobus) productivity: an analysis of the determinants of productivity | 2015 | Acta Horticulturae | https://www.researchgate.net/profile/W_Erskine/publication/231759543_Heritability_and_combining_ability_of_vegetative_and_phenological_characters_of_winged_beans_Psophocarpus_tetragonolobus_L_DC/links/559bcca208ae0035df232f58/Heritability-and-combining-ability-of-vegetative-and-phenological-characters-of-winged-beans-Psophocarpus-tetragonolobus-L-DC.pdf |
| Lorestani B.,Kolahchi N., Ghasemi M., Cheraghi M.,Yousefi N. | Survey the Effect of Oil Pollution on Morphological Characteristics in Faba Vulgaris and Vicia Ervilia | 2012 | Journal of chemical Health Risks | https://kundoc.com/pdf-nodule-formation-and-distribution-during-the-establishment-stage-of-six-selectio.html |
| Zeinab Aboali And Saeed Saeedipour | Efficacy Evaluation of some Herbicides for Weed Management and Yield Attributes in Broad Bean (Vicia faba) | 2015 | Research Journal of Environmental Sciences | https://www.actahort.org/books/1102/1102_9.htm |
| M. J. Kropff, J. Moop, J. Goudriaan,W. Smeets, A. Leemans And C. Kliffen | The effects of long-term open-air fuimigation with so2 on a field crop of broad bean (Vicia faba L.) II. Effects n growth components, leaf area development and elemental composition | 1989 | New phytologist | <http://www.jchr.org/article_543998_fa3f22c0b235522a9a67e4286d9e21cc.pdf> |
| Maé Guinet, Bernard Nicolardot, Cécile Revellin, Vincent Durey, Georg Carlsson, Anne-Sophie Voisin | Comparative effect of inorganic N on plant growth and N2fixation of ten legume crops: towards a better understanding of the differential response among species | 2018 | Plant and Soil | docsdrive.com/pdfs/academicjournals/rjes/0000/72751-72751.pdf |
| S. Nadal, M.T. Moreno, B. Roman | Control ofOrobanche crenata inVicia narbonensisby glyphosate | 2008 | Crop Protection | https://core.ac.uk/download/pdf/29359289.pdf |
| J. Ramirez-Garcia, P. Almendros, M. Quemada | Ground cover and leaf area index relationship in a grass, legume and crucifer crop | 2012 | PLANT SOIL ENVIRON | https://link.springer.com/article/10.1007/s11104-018-3788-1 |
| Anurag Saxena, Y.V. Singh, D.V.Singh And Raj Singh | Weed Management in Mothbean (Vigna aconitifolia) in Arid Region of Rajasthan | 2003 | Annals of Arid Zone | https://sci-hub.tw/https://www.sciencedirect.com/science/article/pii/S026121940700261X |
| R. K. Chopra, K. R. Koundal And Madhu Kansal | Comparative behaviour of seedlings of sorghum and some tropical legumes in relation to leaf expansion and growth | 1985 | J. Agric. Sci. | http://oa.upm.es/16514/1/INVE_MEM_2012_134786.pdf |
| K. Belfry And P. H. Sikkema | Weed management in adzuki bean: a review | 2018 | Canadian Journal of Plant Sciences | http://www.cazri.res.in/annals/2003/2003-June-Vol42-No2-CH4.pdf |
| David W. Emerich, Hari B. Krishnan | Nitrogen Fixation in Crop Production | 2009 | American Society of Agronomy, Crop Science Society of America, Soil Science Society of America | [https://sci-hub.tw/https://www.cambridge.org/core/journals/journal-of-agricultural-science/article/comparative-behaviour-of-seedlings-of-sorghum-and-some-tropical-legumes-in-relation-to-leaf-expansion-and-growth/7269D1A9BF6CD2732817DAAEC345033C](https://sci-hub.tw/https:/www.cambridge.org/core/journals/journal-of-agricultural-science/article/comparative-behaviour-of-seedlings-of-sorghum-and-some-tropical-legumes-in-relation-to-leaf-expansion-and-growth/7269D1A9BF6CD2732817DAAEC345033C) |
| M.U. Kulsum, M.A. Baque And M.A. Karim | Effects of Different Nitrogen Levels on the Leaf Chlorophyll Content Nutrient Concentration and Nutrient Uptake Pattern of Blackgram | 2007 | Pak. J. Bot. | http://www.nrcresearchpress.com/doi/full/10.1139/cjps-2018-0026 |
| Shalini Singh, S.B.Agrawal, Poonamsingh, M.Agrawal | Screening three cultivars of Vigna mungo L. against ozone by application of ethylenediurea (EDU) | 2010 | Ecotoxicology and Environmental Safety | https://books.google.fr/books?id=DjkFPiPP-BkC&dq=vigna+mungo+%25ndfa&hl=fr&source=gbs_navlinks_s |
| D.B.Ishaya, P.Tunku, M.S.Yahaya | Effect of pre-emergence herbicide mixtures on cowpea (Vigna unguiculata (L.) Walp) at Samaru, in northern Nigeria | 2008 | Crop Protection | <https://scialert.net/fulltextmobile/?doi=pjbs.2007.250.254> |
| M. A. Hossain*, M. A. Hasan, S. Sikder And A. K. M. M. B. Chowdhury | Leaf Characteristics and Yield Performance of Mungbean (Vigna radiata L.) Varieties under Different Levels of Shading | 2017 | The agriculturists |  |
| Melese Lema,Bililign Mekonnen And Getachewu Gudero | Performance and Growth Analysis of Three Mungbean (Vigna Radiate (L.) Wilczek) Genotypes at Hawassa, Ethiopia | 2018 | international Journal of Horticulture & agriculture | https://www.sciencedirect.com/science/article/pii/S026121940700316X |
| Ruben Milla | Com. Pers |  |  |  |
| Passarinho, J. A., Rodrigues, M. L., Osório, M. L., Ricardo, C. P. P., & Chaves, M. M. | Physiological responses of lupinus mutabilis to phosphorus nutrition and season of growth. | 2000 | Journal of Plant Nutrition, | :http://dx.doi.org/10.15226/2572-3154/3/2/00118 |
| Hossein Zakeri, Jeff Schoenau, Albert Vandenberg, Mohammadreza Tayfeh  Aligodarz, And Rosalind A. Bueckert | Indirect Estimations of Lentil Leaf and Plant N by SPAD Chlorophyll Meter | 2015 | International Journal of Agronomy |  |


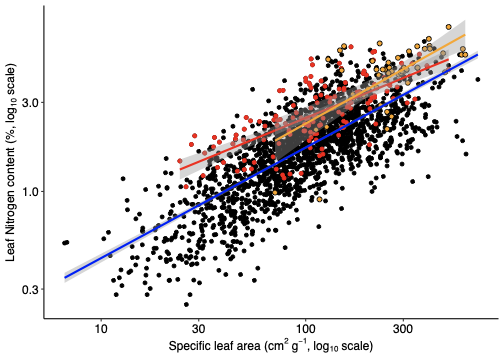


Supplementary figure S1. Relationship between specific leaf area and leaf nitrogen content across wild species (Wright et al. 2004; black dots), nitrogen fixing species (Wright et al. 2004; red dots) and nitrogen-fixing pulses species (this study; orange dots). Lines are linear regression lines for each dataset, respectively.


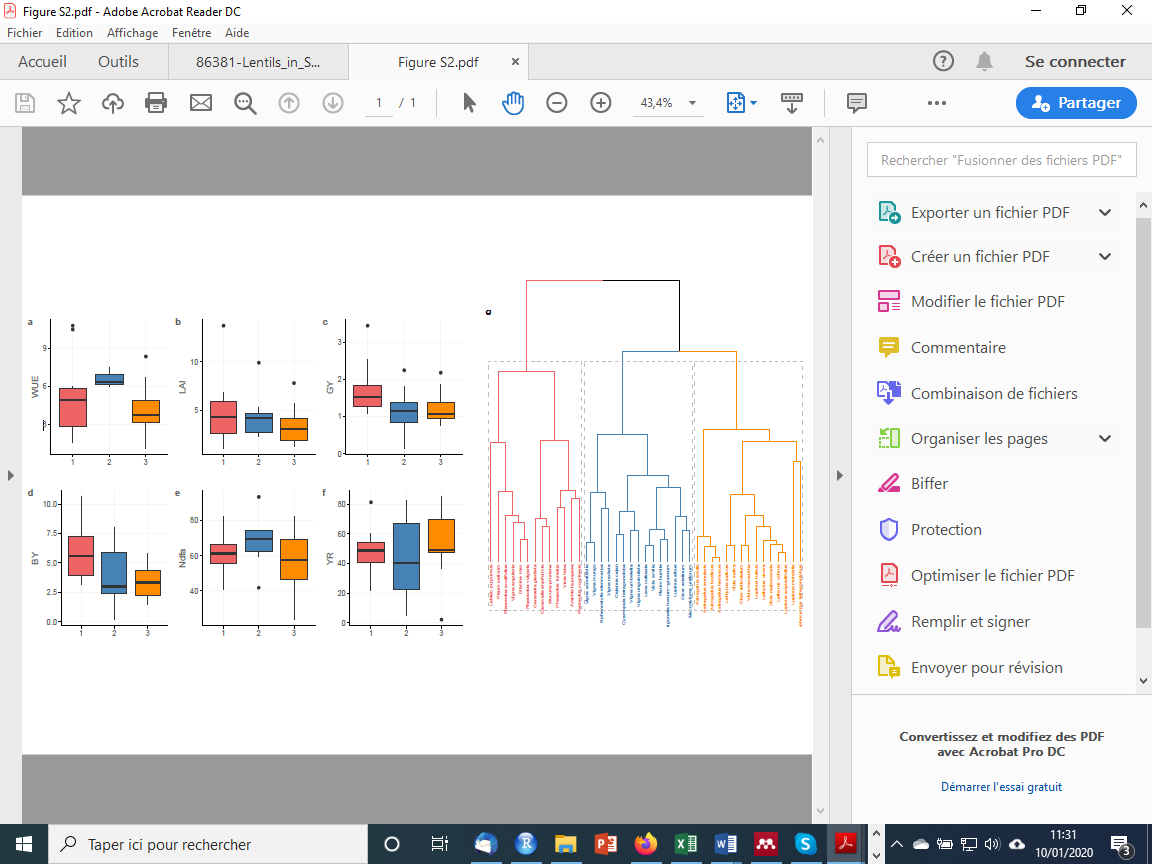


**g**

Supplementary figure S1: Cluster’s values over each property considered: water use efficiency kg ha^-1^ mm^-1^ (a), leaf area index (b), grain yield t ha^-1^(c), biomass yield t ha^-1^(d), nitrogen derived from atmosphere % (e), yield reduction due to weeds % (f) and hierarchical clustering of the 43 pulses species based on the five most important traits for properties predictions (SLA, LNC, TSW, DM, leaflet length) (g)
